# GPCR Modulation of Thieno[2,3-*b*]pyridine Anti-Proliferative Agents

**DOI:** 10.3390/molecules22122254

**Published:** 2017-12-18

**Authors:** Ayesha Zafar, Suat Sari, Euphemia Leung, Lisa I. Pilkington, Michelle van Rensburg, David Barker, Jóhannes Reynisson

**Affiliations:** 1 School of Chemical Sciences, University of Auckland, 23 Symonds Street, 1142 Auckland, New Zealand; ash_imran@hotmail.com (A.Z.); lisa.pilkington@auckland.ac.nz (L.I.P.); m.vanrensburg@auckland.ac.nz (M.v.R.); d.barker@auckland.ac.nz (D.B.); 2 Faculty of Pharmacy, Hacettepe University, 06100 Ankara, Turkey; suat1039@gmail.com; 3 Auckland Cancer Society Research Centre and Department of Molecular Medicine and Pathology, University of Auckland, 1142 Auckland, New Zealand; e.leung@auckland.ac.nz

**Keywords:** docking, molecular and homology modelling, drug discovery, chemical space

## Abstract

A panel of docking scaffolds was developed for the known molecular targets of the anticancer agents, thieno[2,3-*b*]pyridines, in order to glean insight into their mechanism of action. The reported targets are the copper-trafficking antioxidant 1 protein, tyrosyl DNA phosphodiesterase 1, the colchicine binding site in tubulin, adenosine A2A receptor, and, finally, phospholipase C-δ1. According to the panel, the A2A receptor showed the strongest binding, inferring it to be the most plausible target, closely followed by tubulin. To investigate whether the thieno[2,3-*b*]pyridines modulate G protein-coupled receptors (GPCRs) other than A2A, a screen against 168 GPCRs was conducted. According to the results, ligand **1** modulates five receptors in the low µM region, four as an antagonist; CRL-RAMP3 (IC_50_—11.9 µM), NPSR1B (IC_50_—1.0 µM), PRLHR (IC_50_—9.3 µM), and CXCR4 (IC_50_—6.9 µM). Finally, one agonist, GPRR35, was found (EC_50_ of 7.5 µM). Molecular modelling showed good binding to all of the receptors investigated; however, none of these surpass the A2A receptor. Furthermore, the newly-identified receptors are relatively modestly expressed in the cancer cell lines most affected by the thieno[2,3-*b*]pyridines, making them less likely to be the main targets of the mechanism of action for this compound class. Nevertheless, new modulators against GPCRs are of an interest as potential hits for further drug development.

## 1. Introduction

Reynisson et al. [1] have established that the class of thieno[2,3-*b*]pyridines (TPs) have potent anticancer activity against a variety of tumour cell lines. Their activity was discovered by the virtual high throughput screen (vHTS) against the phospholipase C-γ2 (PLC-γ2) isoform, a potential molecular target for anticancer therapy [2]. Testing against the National Cancer Institute’s human tumour cell line panel (NCI-60) [3] revealed that the most potent analogues had low nano-molar growth inhibition, particularly against breast and melanoma tumour cell lines, but also against other cancer types [4,5,6,7].

**Figure 1:**
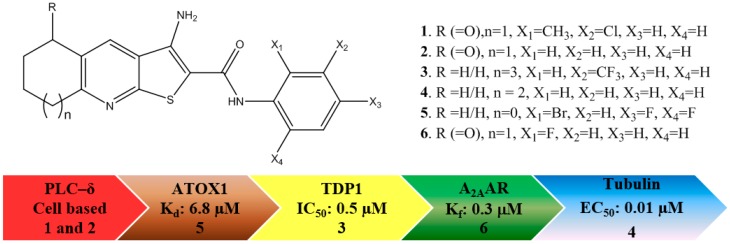
The molecular structures of the active thieno[2,3-*b*]pyridines (TP) derivatives and their established cancer related targets.

TPs are reported to modulate a number of molecular targets that are implicated in cancer progression: (i) phospholipase C-δ1/3 (PLC), deduced by the same cellular behaviour of the MDA-MB-231 breast cancer cell line upon administration of TP and the knockout (silencing) of the PLC-δ1/3 genes; [8,9] (ii) copper-trafficking antioxidant 1 (ATOX1) protein, the inhibition of which reduces the proliferation of cancer cells [10]; (iii) tyrosyl DNA phosphodiesterase 1 (TDP 1), a phospholipase D enzyme, involved in repairing DNA damage [11]; (iv) the colchicine binding site in tubulin [12,13], an established target for anticancer drugs; and (v) adenosine A_2A_ receptor (A_2A_AR) [14], a G protein-coupled receptor (GPCR) implicated in the immune response to tumours [15]. The reported targets are shown in Figure 1, along with the most potent measured inhibition value and the corresponding TP molecular structure.

**Figure 2:**
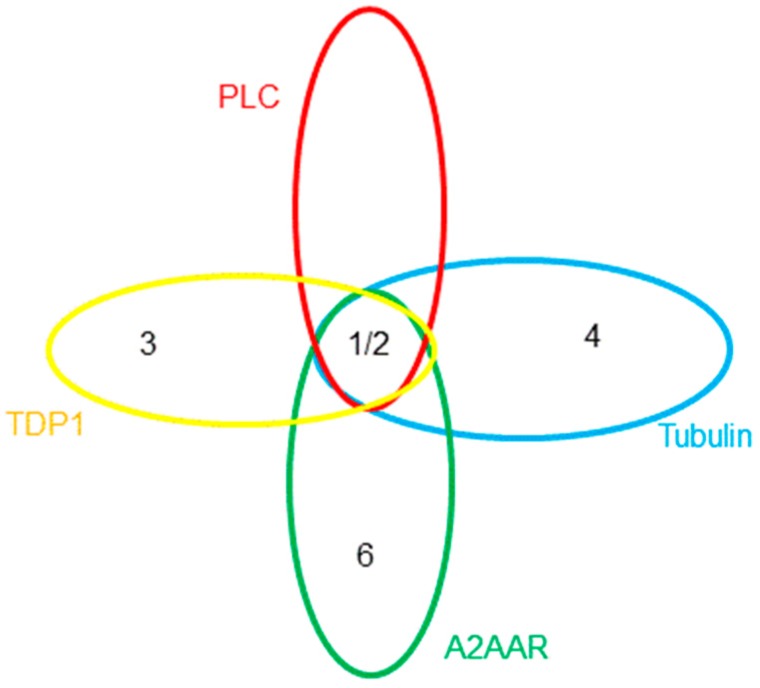
The possible location of the potent TP derivatives in chemical space relevant to the volumes of activity for each of the targets. Copper-trafficking antioxidant 1 (ATOX1) and analogue **5** are omitted for clarity.

To complicate the interpretation of this data, different assay formats are used for each of the targets. Also, various derivatives of the TPs give the best response, i.e., **1** and **2** give excellent overall potency in cell-based assays pointing to PLC-δ inhibition, **3** gives the most favourable IC_50_ for TDP1, **4** has the best tubulin inhibition profile, whereas **5** has the lowest reported K_d_ against ATOX1, and, finally, **6** is the most active analogue for A_2A_AR. Since these derivatives were discovered by different research groups, not using the same compound collections and synthetic strategies, it is not surprising that various derivatives of TP were discovered and developed. Furthermore, each molecular target should have a unique structure-activity relationship, resulting in different TP derivatives being tested. When this is considered in terms of chemical space, Figure 2 can be produced to map locations of the active derivatives, in which each ellipse represents the active volume of chemical space for each target. Taking into account the available data, an extensive overlap between the aforementioned targets can be envisioned. It is currently not known which receptor/enzyme’s modulation produces the pronounced anticancer potential of the TPs or indeed whether a synergistic effect is responsible for the efficacy. For the further development of the TPs as potential clinical candidates, it is vital to elucidate the molecular mechanism responsible for its potency. 

The aim of this study is to glean insight into the molecular mechanism responsible for the anticancer potential of the TPs by conducting molecular modelling against the reported targets. The predicted binding affinities should give an indication as to which experiments are most likely to shed light on the mode of action of this class of anticancer compounds. 

## 2. Results

A docking panel of the putative bio-molecular targets of the TPs was developed consisting of PLC-δ1, ATOX1, TDP1, tubulin’s three binding sites, and the A_2A_AR GPCR. The aim was to compare the scores of the docked TPs derivatives in order to establish which target is most likely to be modulated, thus leading to the observed anticancer effects.

### 2.1. PLC-δ1

**Figure 3:**
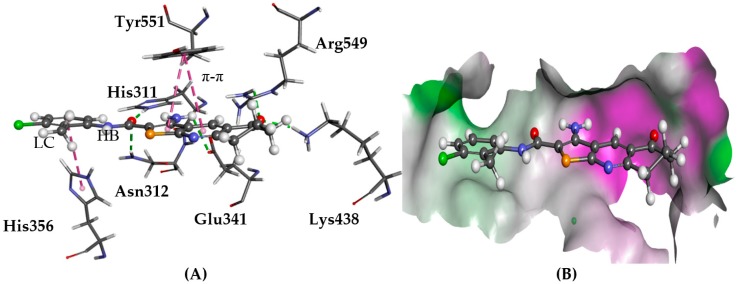
The docked conformation of **1** in the binding site of PLC-δ1 using ChemPLP. (**A**) Hydrogen bonds (HB) are depicted as green dotted lines between ligand and the amino acids Asn312, Lys438, Glu341, Arg549, His311, and π-π stacking, with Tyr551 shown with dashed purple lines; (**B**) **1** shown in the binding pocket with the protein surface rendered. Purple represents hydrogen bond donor regions and green depicts hydrogen bond acceptor regions.

There is evidence for PLC-δ1 supporting the growth and migration of neoplastic mammary epithelial cells, linking this target to cancer growth [16]. Upon docking to PLC-δ1, derivatives **1**–**6** were predicted to have the same/similar binding modes previously reported using both GOLD and Glide [1,4,5,11]. Similar poses were generated with consistent interactions and strong affinity, as well as good overlap with the co-crystallised inositol 1,4,5-triphosphate. Hydrogen bonds with Asn312, Lys438, and His311 were observed as well as the side chains of Glu341, Tyr551, and Gly554, as shown in Figure 3.

### 2.2. ATOX1

**Figure 4:**
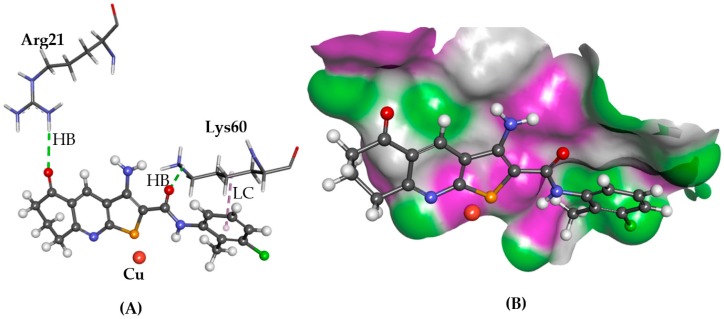
The docked conformation of **1** to the binding site of ATOX1 using GS. (**A**) Hydrogen bonds (HB) are depicted as green dotted lines between **1** and the amino acid Arg21. Furthermore, a lipophilic contact (LC) is shown as purple dashed lines between Lys60 and derivative **1**; (**B**) **1** shown in the binding pocket with the protein surface rendered. Purple represents hydrogen bond donor regions and green depicts hydrogen bond acceptor regions.

The ATOX1 copper trafficking protein is linked to reduced proliferation of cancer cells demonstrated by knockout experiments of its gene [10]. Compound **6** is reported to form hydrogen bonds with the side chains of Glu17, Arg21, and Lys 60 amino acid residues, blocking the protein-protein interface of ATOX1 for copper ion delivery [10]. In the case for **1** and **2** using GOLD, similar poses were predicted as those reported. Compound **1** blocked the copper-trafficking interface by forming a hydrogen bond with Arg21 and lipophilic contacts with Ala18 and Lys60, as shown in Figure 4, whilst **2** displayed hydrogen bonding interactions with Cys15 and Thr58, as well as a lipophilic contact between the phenyl and Lys60. Glide predicted a binding mode for **6** in which its thieno[2,3-*b*]pyridine moiety is positioned between Glu17, Arg21, and Lys60 side chains, with a hydrogen bond with the Glu17 side chain via its amino group and with the Cys12 side chain with the amide NH moiety. Cys12 and Cys15 are two of the four cysteine residues, which coordinate with a copper ion at the interface of two protein chains of ATOX1, thus blocking access to these residues inhibiting ion transfer. In the case of **1**, however, a different binding pose was observed with the phenyl ring close to Lys60 and formed a hydrogen bond with Arg21. 

### 2.3. TDP1

**Figure 5:**
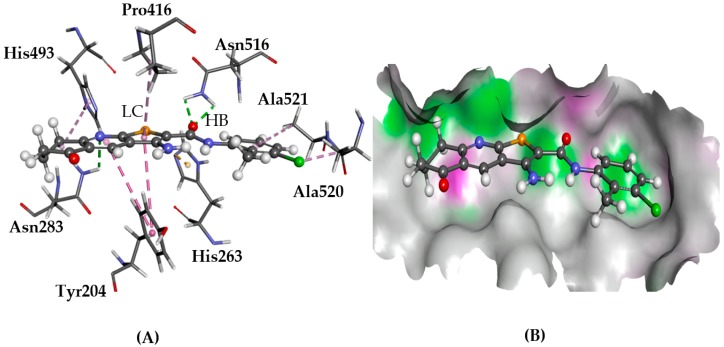
The docked conformation of **1** to the binding site of TDP1 using ChemPLP. (**A**) The hydrogen bonds (HB) are depicted as green dotted lines between ligand and the amino acids Asn283 and Asn516. Furthermore, lipophilic contacts (LC) are shown as purple dashed lines; (**B**) **1** shown in the binding pocket with the protein surface rendered. Purple represents hydrogen bond donor regions and green depicts hydrogen bond acceptor regions.

Camptothecin and its analogues (e.g., topotecan and irinotecan) constitute an effective and widely used class of cancer therapeutics. They work by inhibiting the enzyme DNA topoisomerase I (topo I), preventing DNA replication. In human cells, TDP1 is important to the mechanism for removing DNA lesions [17,18]. In cancer cells, however, TDP1 counters the action of camptothecin by repairing drug-created stalled topo I-DNA complexes, thus making TDP1 a potential anticancer target [19,20]. For TDP1, both His263 and His493 are proposed to play a key role in its biological function and, as such, the binding pocket is defined at these amino acids [21,22,23]. When docked to TDP1 using GOLD, all the ligands fitted into the pocket in a similar way as those depicted for **1** in Figure 5. These results are in line with previously published modelling data for the TPs [11]. Glide predicted a good fit for all the ligands, despite some variations in hydrogen bonding patterns, as for the GOLD predictions. The main difference between the Glide- and GOLD-predicted poses was the reverse orientations of the phenyl and thieno[2,3-*b*]pyridine rings.

### 2.4. Tubulin

Tubulin-targeting agents are widely used for the treatment of cancer. They disrupt/inhibit tubulin dynamics by acting as stabilisers or destabilisers. Three different binding sites have been identified for targeting microtubules, namely colchicine-, taxol-, and *Vinca* alkaloid-binding pockets [24].

In order to establish the most plausible binding pocket for the TPs, **1**–**6** were docked to the three identified sites and the results are given in Table 1. A clear trend is seen for the colchicine site predicted for the GS and Glide values, with the ChemPLP results also leaning towards this site. CS and ASP gave similar scores for all three sites. Furthermore, the results are in line with earlier findings where TP derivatives have been predicted to bind to the colchicine site [12,13].

**Figure 6:**
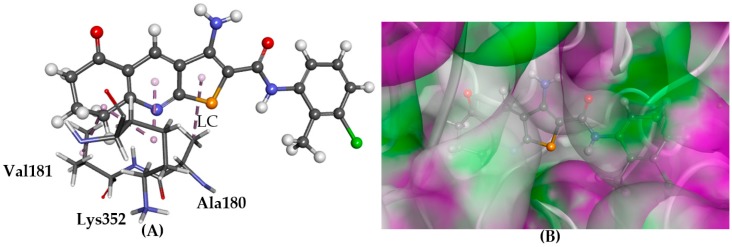
The docked conformation of **1** to the binding site of Tubulin-colchicine using ChemPLP. (**A**) Lipophilic contacts (LC) are shown as purple dashed lines; (**B**) **1** shown in the binding pocket with the protein surface rendered. Purple represents hydrogen bond donor regions and green depicts hydrogen bond acceptor regions.

According to the GOLD docking results, compounds **1**–**6** have a similar predicted configuration in the colchicine-binding site and showed stacking/polar interactions with tubulin (Figure 6), sitting deep in the hydrophobic pocket (Ser178, lipophilic contacts with Ala180 and Val181). Docking results with Glide were highly consistent for TPs **1**–**6**, yet somewhat different from those produced by GOLD. The binding affinities of the Glide predictions were high, and a common hydrogen bond with Val181 backbone NH, also a feature of co-crystallised colchicine, was observed.

### 2.5. Testing for GPCRs Activity

One of the reported targets for the TPs is the A_2A_AR GPCR receptor with nano-molar activity [14]. It is estimated that 30–50% of all drugs on the market target GPCR receptors [25]. It was therefore decided to investigate whether the TPs modulate other GPCR receptors. 

Derivative **1** was screened against the gpcrMAX Panel containing 168 receptors in both agonist and antagonist formats at 10 µM by the DiscoverX company [26]. The analysis of the data revealed five potential antagonist receptors and one agnostic receptor. In brief, ligands where considered as potential antagonists with inhibition >35% and agonists with activity >30% (see Tables S8–S10 in Supplementary Materials). These six receptors were tested for dose-response to quantify their effect and five targets (four antagonists/one agonist) were active; the results are shown in Table 2. The receptor, HTR1B, showed activity in the primary screen but had IC_50_ > 50 µM for the dose-response and was therefore not considered to be a hit. The dose response curves are given in the Supplementary Information pages 15 and 16. Furthermore, the complete technical reports from the company DiscoverX containing controls are available in the Supplementary Information.

The five receptors are modulated by TP **1** in the low µ-molar region and are therefore less potent than A_2A_AR (ADORA2A), the known GPCR, which was unfortunately not in the screening panel. Interestingly, a related Adenosine A3 (ADORA3) receptor was in the panel and some activity was observed (25%) in the antagonist format, but not enough to be taken forward for quantitative testing. Recently, a panel of TPs was tested for in vitro anti-platelet activity and showed both platelet activation and aggregation [27]. The authors concluded that the TPs are potential inhibitors for the P2Y_12_ receptor, the target for clopidogrel, the standard clinical therapeutic. The P2Y_12_ receptor (P2RY12) had 13% agonistic activity according to the assay, but no antagonist modulation was observed. 

The activity of **1** against the identified targets was relatively modest, in the low μ-molar range (see Table 2), which implies that the low nano-molar activity seen against some tumour cell lines cannot be explained by the modulation of these receptors. Nevertheless, identifying activity against any GPCRs is helpful in elucidating their mechanisms of action and in the development of potent small molecules against specific receptors. 

Molecular modelling was performed in order to analyse and compare the binding mode of the TPs against A_2A_AR and the identified receptors. A_2A_AR and CXCR4 both have published crystal structures [28,29], and CRL-RAMP and GPR35 have published homologies models [30,31]. Protein structures for NPSR1B and PRLHP were obtained from the GPCR database [32].

#### 2.5.1. A_2A_AR

The A_2A_AR receptor is linked to the immune response of tumours by T regulatory cell function, suppression and modulation of natural killer cell cytotoxicity, and tumour-specific CD4+/CD8+ activity [15]. In the case of A_2A_AR antagonists, a common binding motif involves π-π stacking between aromatic moieties of the ligands and the Phe168 side chain, as well as hydrogen bonding interactions with Asn253 and Glu169 side chains [14].

**Figure 7:**
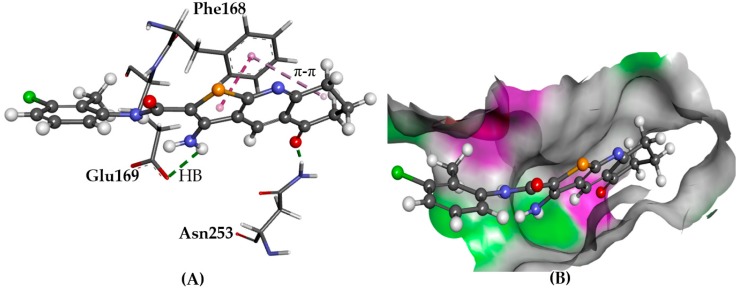
The docked conformation of **1** to the binding site of A_2A_AR using ChemPLP. (**A**) Hydrogen bonds (HB) are depicted as green dotted lines between ligand and the amino acids Asn253 and Glu169. π-π stacking with Phe168 is shown with dashed purple lines; (**B**) **1** shown in the binding pocket with the protein surface rendered. Purple represents hydrogen bond donor regions and green depicts hydrogen bond acceptor regions.

GOLD and Glide predicted very similar binding poses for compounds **1**–**6**. Key interactions such as hydrogen bonding with Asn253 and Glu169 side chains and π-π stacking with the Phe168 aromatic side chain were common among the derivatives, as were high binding affinities (Figure 7).

#### 2.5.2. CXCR4

CXCR4 is a chemokine receptor involved in cancer metastasis and inflammatory diseases, as well as HIV-1 infection [29]. Interestingly, modulation of this receptor with small organic ligands has been reported for both metastasis and inflammation [33].

**Figure 8:**
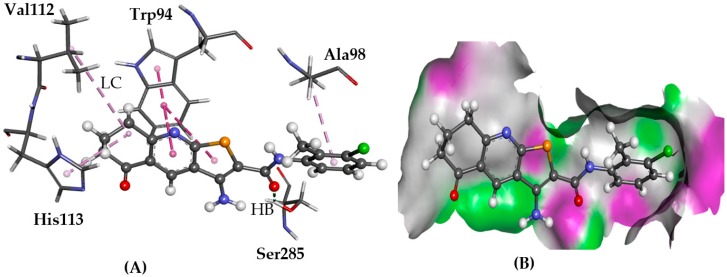
The docked conformation of **1** to the binding site of CXCR4 using ChemPLP. (**A**) A hydrogen bond (HB) is depicted as a green dotted line between **1** and the amino acids Ser285. Lipophilic contacts (LC) are predicted with Trp94 shown with purple dashed lines; (**B**) **1** shown in the binding pocket with the protein surface rendered. Purple represents hydrogen bond donor regions and green depicts hydrogen bond acceptor regions.

GOLD predicted hydrogen bonding between Try94 and Asp97 side chains via the amino group of the TPs as seen for **1** in Figure 8. Also, π-π stacking with Trp94 side chain was observed for some compounds with the thieno[2,3-*b*]pyridine ring. Glide docking was very consistent in terms of binding poses, which were in agreement with the GOLD predictions and the co-crystallised conformation of ITD ((6,6-dimethyl-5,6-dihydroimidazo [2,1-*b*] [1,3]thiazol-3-yl)methyl *N*,*N*′-dicyclohexylimidothiocarbamate).

#### 2.5.3. CRL-RAMP3

Calcitonin receptor-like activity modifying protein 3 (CRL-RAMP3), is a member of the RAMP family of receptors, single transmembrane-domain proteins. CRL-RAMP3 regulates calcitonin and is a linear polypeptide hormone involved in the calcium ion regulation in blood [34]. The CRL-RAMP3 complex has been indicated to be important to angiogenesis and therefore to tumour growth [35]. A published homology model of RAMP3 was used for the modelling [30]. The model of RAMP3 suggests that Tyr84 can contact the Try52 of adrenomedullin (AM) and calcitonin gene-related peptides (CGRP) Phe37 phenyl rings and is therefore expected to decrease the potency of both peptides at the AM2 receptor [36,37]. Furthermore, Glu74 and Tyr84 may interact with ligands that bind to the CRL-RAMP3 heterodimer.

**Figure 9:**
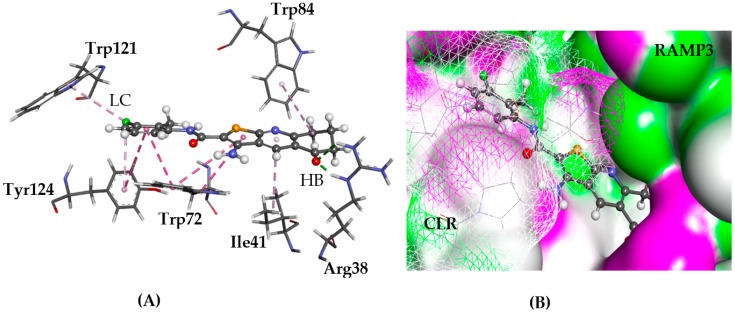
The docked conformation of **1** to the binding site of CLR-RAMP3 using ChemPLP. (**A**) A hydrogen bond (HB) is depicted as green dotted line between ligand and the amino acid Arg38. Tyr52 and Trp84 are in lipophilic contact (LC) with the ligand shown with dashed purple lines; (**B**) **1** is shown in the binding pocket with the protein surface rendered and seems to fit in the cleft between CRL and RAMP3. Purple represents hydrogen bond donor regions, and green depicts hydrogen bond acceptor regions.

The GOLD and Glide docking predicted similar poses for all ligands, which occupied the cleft between CRL and RAMP3. They were observed to π-π stack with Trp72 and Tyr124 aromatic side chains of CRL. The thieno[2,3-*b*]pyridine moiety was surrounded by RAMP3 residues Glu74, Trp84, and Arg38 (Figure 9).

#### 2.5.4. GPR35

G protein-coupled receptor 35 (GPR35) is important to the tryptophan metabolic pathway, acting as the receptor for the intermediate kynurenic acid. The homology model of human GPR35 (hGPR35) with bufrolin docked in the active site was provided by McKenzie et al. [31]. Re-docking of bufrolin resulted in relatively low RMSD values with an average of 2.3 Å, indicating good reproducibility. 

Docking of the TPs using GOLD yielded plausible binding poses. Hydrogen bonds were predicted with Arg239 side chain amide NH_2_ via the carbonyl on cycloalkane and with Arg148 via NH_2_ on the thiophene ring. The TPs are predicted to form π-π stacking with Phe147.

**Figure 10:**
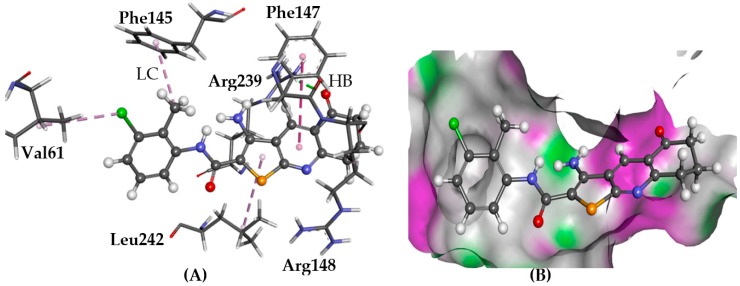
The docked configuration of **1** to the binding site of GPR35 using PLP scoring function. (**A**) Hydrogen bonds (HB) are depicted as green lines between the ligand and the amino acid Tyr243 and Arg239. The π-π stacking is observed with Phe147 shown with a purple dashed line as well as lipophilic contact (LC) with Val61, Phe145, and Leu242; (**B**) the protein surface is rendered. The bicyclic group is inserted in a lipophilic pocket. Purple represents hydrogen bond donor regions and green depicts hydrogen bond acceptor regions.

Glide docking predicted two different binding trends for **1**–**6**. Compounds **1** (Figure 10), **3**, and **6** aligned in a similar way as was observed with GOLD, forming hydrogen bonds with Asn230 and Arg239 side chains. Alternatively, **2**, **4**, and **5** formed two hydrogen bonds; one with the Cys146 backbone and one with the Ser246 side chain.

#### 2.5.5. NPSRb1

**Figure 11:**
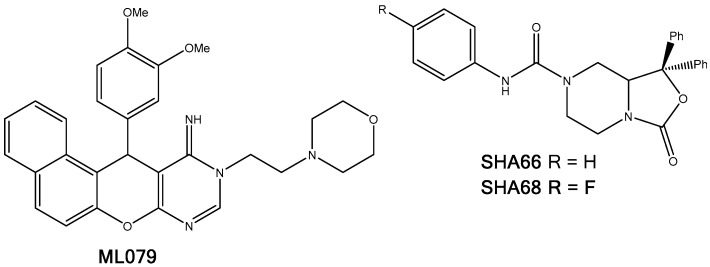
Molecular structures of the known NPSRb1 antagonists, ML079, SHA66, and SHA68.

Neuropeptide S receptor b1 (NPSRb1) is linked to bronchial smooth muscles and asthma as well as inflammatory bowel disease [38]. Two residues in the second transmembrane region (TM2) of human NPSRb1, namely Asn107 and Asp105, are reported to be important for antagonist affinity [39,40]. Furthermore, three known antagonists of NPSRb1, with nano-molar activities, are known (Figure 11) [41,42]. This information was used to locate the binding site of the receptor and to assess which of the amino acid residues are important for a successful binding.

**Figure 12:**
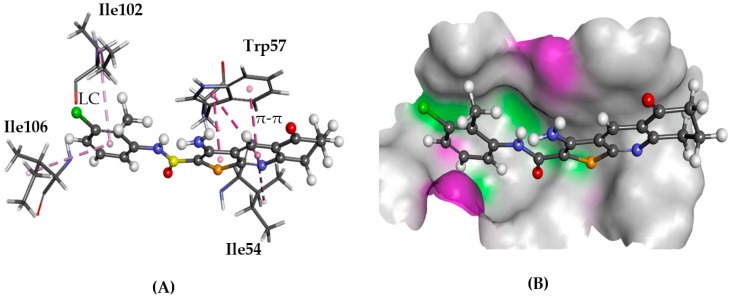
The docked configuration of **1** to the binding site of NPSRb1 using GS scoring function. (**A**) π-π stacking and lipophilic contact (LC) are depicted as purple lines between the ligand and the amino acids; (**B**) **1** shown in the binding pocket with the protein surface rendered. Purple represents hydrogen bond donor regions, and green depicts hydrogen bond acceptor regions.

Docking the known antagonists in the vicinity of the target residues yielded plausible binding poses. The TPs almost perfectly overlapped with the predicted poses of known antagonists, SHA66 and SHA68. The phenyl ring was inserted into the cleft between two TM domains and parallel to helices, just like the *p*-fluorophenyl moiety of SHA68 and the TP moiety was in contact with the extracellular domain residues (Figure 12). In this conformation, the carbonyl group formed hydrogen bonds with the amino group of Ile106.

#### 2.5.6. PRLHR

Prolactin releasing hormone receptor (PRLHR) has seven transmembrane domains. Prolactin is a protein hormone involved in lactation of female mammals. There is some evidence that it increases the risk of breast cancer [43]. To our knowledge, no biological or theoretical information regarding binding site for PRLHR ligands exists. Therefore, possible binding site cavities were determined using Site Map followed by docking of **1**–**6** to the identified cavities on the homology model. Docking results obtained from the cavity, which was detected at the centre of TM2-6 and close to extracellular loops, were reasonably consistent for both GOLD and Glide.

**Figure 13:**
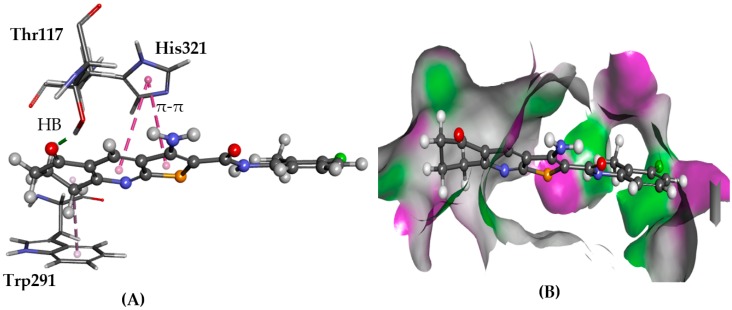
The docked configuration of **1** to the binding site of PRLHR using GS scoring function. (**A**) A hydrogen bond (HB) is depicted as green lines between the ligand and the amino acid Asn298. The π-π stacking is observed with His321 and Trp291 shown as purple dashed lines; (**B**) **1** shown in the binding pocket with the protein surface rendered. Purple represents hydrogen bond donor regions, and green depicts hydrogen bond acceptor regions.

In the binding mode of **1** obtained from GOLD, a hydrogen bond with Asn298 was observed and π-π stacking with His321 (Figure 13). Compound **2** formed hydrogen bonds with Asn298 and Thr117, and π-π stacking with Phe138 and Trp291. Glide predicted similar binding for **1**, **2**, **4**, and **5**, with consistent interactions with the putative binding site residues. They had hydrogen bonding with Asn298 side chain donor via their amide carbonyl and π-π stacking with Trp291 and His295 side chains with the phenyl ring of TS.

## 3. Discussion

As **1**–**6** were docked using five different scoring functions, an estimate was generated to identify which molecular targets are most likely to be modulated by the TPs. The scoring function results are given for each derivative **1**–**6** in the Supplementary Information. The results, for the six ligands, were averaged and the standard deviation was derived (Table 3).

Looking at the results, for GS A_2A_AR has the highest score, closely followed by tubulin, CXCR4, and GPR35, while the other targets have clearly lower predicted scores. CS again predicts A_2A_AR to have the highest score, with the second highest shared by the rest of the GPCRs, except NSPRb1. The ChemPLP scoring function put A_2A_AR as the top target, followed by CXCR4 and CRL-RAMP3. ChemPLP has been shown to be the best, or one of the best, performing scoring functions available [44,45]. ASP predicted A_2A_AR to be the best, followed by CXCR4 and GPR35. Finally, Glide predicted A_2A_AR to have the highest affinity with the TPs, followed by tubulin. Based on these results, it is apparent that A_2A_AR is the most likely target, with the highest predicted score for all of the scoring functions used. 

The results for all the scoring functions were normalised to the highest score (A_2A_AR), giving them an equal weight. They were then summed and divided to give the best score of 10 (Total column in Table 3) and standard deviations were derived. When these numbers were analysed, it was seen that the GPCRs were predicted to be modulated, except NSPRb1, with tubulin also obtaining a good score, followed by PLC-δ1. According to this analysis, the TPs mostly exert their anticancer effect by GPCR modulation.

As can be seen in Figure 1, the TPs derivatives do not have radically different chemical structures; however, when the scoring data is analysed, notable shifts in predicted affinities are observed, e.g., removing the substituents on the phenyl ring from **1** resulting in **2** increases the affinity of the latter towards PLC-δ1 for both the GS and PLP scoring functions (see Tables S2–S7 in the Supplementary Materials). This means that each modification of the TPs changes the affinity to many of the targets, rendering it very difficult to generate a reliable structure activity relationship (SAR) for cell-based data. Thus, it is a multidimensional problem to optimise the TPs’ structure for anticancer activity. The changes in water solubility and, consequently, cell penetration, between the derivatives also needs to be considered, adding further complexity to the problem.

### Transcript Levels in the Cancer Cells Affected by TPs

The NCI60 transcriptome database and Cellminer tools [46] (discover.nci.nih.gov/cellminer) were used to assess the expression of *mRNA* of the identified targets in the National Institute of Cancer human tumour cell lines panel (NCI-60) [3]. The *z*-score representations, a measure of *mRNA* transcript intensity [46], were collected for all the available targets and the results are given in the Supplementary Materials. Unfortunately, CRL-RAMP3 and PRLHR were not available in the database. The melanoma cell line MDA-MB-435 is the most susceptible tumour cell for the TPs in the NCI-60 panel and the *z*-scores for the targets are given in Table 4, as well as the minimum and maximum values given for the cell line panel.

According to the values in Table 4, only PLC-δ1 and ATOX1 have positive values, i.e., significant *mRNA* is expressed in this cell line making these targets plausible. TDP1 is expressed to some level but TDP1’s expression levels are expected to rise under stress, leading to DNA damage (such a chemotherapy) to aid the cell to survive. Also, tubulin is heavily expressed during mitosis, making it abundant during cell duplication in all of the cancer cell lines. In general, the transcript intensity of the GPCR receptors is low, in particular for CXCR4, but GPR35, A_2A_AR, and NSPRb1 are expressed to some extent.

For further analysis, the expression levels of each target were considered and correlated with the GI_50_ values of ligand **1,** as shown in Tables S11–S20 in the Supplementary Materials.

In general, the data suggest that gene expression level is necessary, but not sufficient, to predict the efficacy of inhibition. *mRNA* abundance and consequent protein expression are known to correlate poorly due to complex biological and technical issues [47].

## 4. Materials and Methods 

### 4.1. Molecular Modelling

Using GOLD [48] and Glide [49] software suites, the compounds were docked to the crystal structure of PLC-δ1 (PDB ID: 1DJX, resolution 2.3 Å) [50], ATOX1 (PDB ID: 1FEE, resolution 1.8 Å) [51], TDP1 (PDB ID: 1MU7, resolution 2.0 Å) [21], A_2A_AR (PDB ID: 3EML, resolution 2.6 Å) [28], and CXCR4 (PDB ID: 3ODU, resolution 2.5 Å) [29], which were obtained from Protein Data Bank (PDB) [52,53]. To rationalise the mechanistic action of the TPs to tubulin, they were docked to the tubulin crystal structures of the three main binding sites: colchicine, *Vinca* alkaloid, and taxol with the respective PDB IDs of 4O2B (resolution 2.3 Å) [54], 4EB6 (resolution 3.47 Å) [55], and 1JFF (resolution 3.5 Å) [56]. The centres of the binding pockets were defined as the position of the position of the Ca^2+^ ion (*x* = 78.99, *y* = 38.878, *z* = −19.069) for PLC-δ1, the sulfur of Cys12 (*x* = 66.121, *y* = −55.634, *z* = 36.866) for ATOX1, the position of the tungsten (W) of the co-crystallised ligand (*x* = 8.312, *y* = 12.660, *z* = 34.452) for TDP1, the co-crystallised ligand centre point (*x* = −9.420, *y* = −9.544, *z* = 56.644) for A_2A_AR, and the centroid of the co-crystalised ligand (*x* = 20.266, *y* = −6.133, *z* = 72.241) for CXCR4. For tubulin, the centre of co-crystallised colchicine (*x* = 13.222, *y* = 8.371, *z* = −23.331), taxol (*x* = 2.182, *y* = −15.848, *z* = 15.866), and vinblastine (*x* = 13.391, *y* = 90.610, *z* = 103.739) were used. 

For the docking studies using GOLD, 50 docking runs were allowed for each ligand with default search efficiency (100%). The GoldScore (GS) [57], ChemScore (CS) [58,59] Piecewise Linear Potential (ChemPLP) [60], and Astex Statistical Potential (ASP) [61] scoring functions were implemented to predict binding modes and relative energies of ligand-receptor complexes. The Scigress ultra version F.J 2.6 program [62] was used to prepare the crystal structures for docking, i.e., hydrogen atoms were added and crystallographic water molecules and co-crystallised ligands were removed. The basic amino acids, lysine and arginine, were defined as protonated. Furthermore, aspartic and glutamic acids were assumed to be deprotonated. The Scigress software suite was also used to build the inhibitors and the MM2 [63] force field was used to optimise the structures.

For Glide, the crystal structures were prepared using Protein Preparation Wizard of Maestro [64], where hydrogens were added, ionisation and tautomeric states were generated by Epik, and proton orientations were set by PROPKA [64]. The same coordinates as those used for GOLD were used for search the space centre in grid generation, and the van der Waals radii of non-polar receptor atoms (partial charge cut-off 0.25) were scaled down to 0.8 Å. Ligands were docked at extra precision, post-docking minimisation was enabled, Epikstate penalties were added to docking scores, and a maximum of 10 poses were recorded per ligand. 

In order to verify the robustness of the docking protocol, the available co-crystallised ligands were re-docked and their RMSD (Root-Mean-Square Deviation) values generated. The results are shown in Table S1 in the Supplementary Materials, and an adequate to excellent overlap was obtained. 

### 4.2. Homology Modelling

The homology models were prepared with Protein Preparation Wizard of Maestro [64]. For the models of NPRS1B and PRLHR, which were obtained from the GPCR data base [32], restrained force field minimisation was performed with convergence of heavy atoms to an RMSD of 0.5 Å to prevent clashes. The coordinates of N1 of Trp-72 (chain A) side chain for CALCRL-RAMP3, the centroid of docked bufrolin for GPR35, the midpoint of Asp105 and Asn107 for NPSR1B, and the centroid of the predicted cavity by Site Map (Schrödinger, LLC, New York, NY, USA, 2015) [64] for PRLHR were picked as the centres of search spaces for each model.

### 4.3. Biological Testing

Compound **1** was screened against the gpcrMAX Panel using 168 different GPCR receptors, in both agonist and antagonist formats. The assays were performed using the PathHunter beta-arrestin enzyme fragment complementation (EFC) technology at DiscoverX [26]. The full data set and the analysis protocol are given in the Supplementary Materials.

## 5. Conclusions

This work communicates the discovery of activity of TPs against GPCRs, albeit in the low µM range. Finding potent ligands against unexplored GPCRs is very helpful in the elucidation of their nature and is the first step in developing a specific ligand for modulation and possibly a clinical candidate. The modelling data indicates A_2A_AR and tubulin to be the most likely targets of the TPs; however, when protein expression data is considered, other targets such as ATOX1 and PLC-δ1 are evidenced to play a role in the anticancer activity of this class of compounds. It is clear that the TPs are prolific inhibitors/modulators of various targets and can therefore be categorised as a privileged structure in drug discovery [65]. The challenge is to develop the TPs to be specific against the target of interest in order to develop them into viable drug candidates.

## Figures and Tables

**Table 1:** The predicted scores of the TPs with tubulin’s colchicine-, taxol-, and *Vinca* alkaloid binding sites.

	Colchicine					Taxol				Vinca				
No	GS	CS	PLP	ASP	Glide	GS	CS	PLP	ASP	GS	CS	PLP	ASP	Glide
**1**	63.9	31.4	54.9	25.4	−6.7	52.9	30.9	52.6	26.3	59.1	30.6	57.2	30.1	−3.8
**2**	62.5	29.6	61.3	28.0	−6.9	50.8	29.7	50.9	25.5	55.9	29.4	49.3	28.7	−3.1
**3**	63.2	25.9	53.4	27.8	−5.7	57.2	31.5	59.1	29.9	54.3	26.4	52.3	31.8	−4.2
**4**	64.4	28.9	68.1	24.8	−7.4	50.5	29.8	53.2	23.7	49.9	25.2	53.1	27.3	−2.8
**5**	65.7	32.7	63.4	31.9	−5.8	51.8	28.8	51.9	24.8	52.2	27.2	54.4	28.9	−4.1
**6**	62.3	26.4	52.7	28.7	−7.4	51.6	28.0	54.0	26.2	55.1	27.9	52.7	28.2	−4.3

**Table 2:** Quantitative modulating values of compound **1** against the identified G protein-coupled receptors (GPCRs). Antagonists values are given as Inhibition Concentration of 50% (IC_50_) and agonist as half maximal effect concentration (EC_50_).

GPCR	μM	
CRL-RAMP3	IC_50_	11.9
NPSR1B	IC_50_	1.0
PRLHR	IC_50_	9.3
CXCR4	IC_50_	6.9
GPR35	EC_50_	7.5

**Table 3:** Predicted average scores and standard deviations for derivatives **1**–**6** against the identified targets. The best scores are in bold and the second best in italics.

	Targets	GS	CS	PLP	ASP	Glide	Total ^1^
1	PLC-δ1	*56.9 ± 4.3*	28.6 ± 1.8	*63.1 ± 2.7*	35.1 ± 1.3	−3.7 ± 0.8	7.7 ± 0.6
2	ATOX1	41.5 ± 1.8	19.9 ± 1.6	41.2 ± 3.7	16.1 ± 1.7	−2.2 ± 0.3	4.8 ± 0.4
3	TDP1	49.7 ± 0.9	26.8 ± 1.2	48.5 ± 3.7	31.2 ± 1.5	−3.2 ± 0.9	6.6 ± 0.5
4	Tubulin-Colchicine	**63.7 ± 1.3**	29.2 ± 2.7	58.9 ± 6.3	27.8 ± 2.5	−6.7 ± 0.7	8.1 ± 0.6
5	A_2A_AR	**64.2 ± 2.8**	**38.6 ± 3.0**	**69.2 ± 1.9**	**40.4 ± 1.5**	**−8.8 ± 0.4**	**10.0 ± 0.5**
6	CXCR4	**62.6 ± 4.3**	*32.9 ± 1.3*	*66.4 ± 1.4*	*37.4 ± 3.0*	−6.3 ± 0.3	*8.9 ± 0.5*
7	CRL-RAMP3	57.1 ± 1.5	*31.7 ± 1.9*	*64.1 ± 4.3*	36.7 ± 2.1	−5.7 ± 0.1	8.4 ± 0.4
8	GPR35	**62.9 ± 1.9**	*32.2 ± 1.7*	62.6 ± 3.9	*38.4 ± 1.7*	−4.7 ± 0.6	8.4 ± 0.5
9	NSPRb1	49.9 ± 2.1	27.1 ± 1.9	58.1 ± 4.6	31.1 ± 1.5	−4.3 ± 0.5	7.2 ± 0.5
10	PRLHR	*56.7 ± 2.9*	*32.8 ± 3.1*	56.9 ± 3.8	32.9 ± 1.4	−5.5 ± 0.8	8.0 ± 0.5

^1^ Scores normalised to A_2A_ (=10) and corresponding standard deviations.

**Table 4:** The expression levels (*z*-scores) of the targets in the melanoma MDA-MB-435 cell line. Also, the minimum and maximum values are given.

Target	*z*-Score	Min.	Max.
PLC-δ1	0.129	−1.088	−4.413
ATOX1	1.055	−0.905	2.886
TDP1	−0.184	−4.662	1.716
Tubulin	−0.822	−0.880	2.877
A_2A_AR	−0.777	−1.412	2.622
CXCR4	−0.667	−0.687	3.615
GPR35	−0.483	−0.514	4.056
NSPRb1	−0.284	−0.503	6.804

## Supplementary Materials

The following are available online at www.mdpi.com/1420-3049/22/12/2254/s1, Table S1: RMSD values of co-crystallised ligands after docking, Table S2: predicted interaction of thienopyridines (**1**) with bio-molecular targets and their binding energies, Table S3: predicted interaction of thienopyridines (**2**) with bio-molecular targets and their binding energies, Table S4: predicted interaction of thienopyridines (**3**) with bio-molecular targets and their binding energies, Table S5: predicted interaction of thienopyridines (**4**) with bio-molecular targets and their binding energies, Table S6: predicted interaction of thienopyridines (**5**) with bio-molecular targets and their binding energies, Table S7: predicted interaction of thienopyridines (**6**) with bio-molecular targets and their binding energies, Table S8: assay mode: agonist, 10 µM of 1, Table S9: assay mode: antagonist, 10 µM of 1, Table S10: results of the dose response experiments for the receptors, Table S11: average transcript intensity *z* scores (ATOX1), Table S12: average transcript intensity *z* scores (PLC δ1), Table S13: average transcript intensity *z* scores (PLC δ3), Table S14: average transcript intensity *z* scores (TDP1), Table S15: average transcript intensity *z* scores (Tubulin Beta 2B), Table S16: average transcript intensity *z* scores (Tubulin Alpha 1A), Table S17: average transcript intensity z scores (A_2A_AR), Table S18: average transcript intensity *z* scores (CXCR4), Table S19: average transcript intensity *z* scores (GPR35), Table S20: average transcript intensity *z* scores (NPSRB1).
