# Supplementary material for: GPCR Modulation of Thieno[2,3-b]pyridine Anti-Proliferative Agents
Source: Molecules. 2017 Dec 18;22(12):2254. doi: 10.3390/molecules22122254 (PMC6149898; doi:10.3390/molecules22122254)
Supplement: Supplementary file 1 [file molecules-22-02254-s001.pdf]

Supplementary Information

Investigation into the mechanism of action for the thieno[2,3-b]pyridine anticancer compounds. A molecular modelling study

Ayesha Zafar, [1] Suat Sari, [2] Euphemia Leung,[3] Lisa I. Pilkington,[1] Michelle van Rensburg,[1] David Barker[1] and Jóhannes Reynisson[1*]

[1]School of Chemical Sciences, University of Auckland, New Zealand

[2]Faculty of Pharmacy, Hacettepe University, Ankara, Turkey

[3]Auckland Cancer Society Research Centre and Department of Molecular Medicine and Pathology, University of Auckland, New Zealand

1. Table S1.RMSD values of co-crystallised ligands after docking. 4

2. Table S2. Predicted interaction of thienopyridines (1) with bio-molecular targets and their binding energies. 4

3. Table S3. Predicted interaction of thienopyridines (2) with bio-molecular targets and their binding energies. 5

4. Table S4. Predicted interaction of thienopyridines (3) with bio-molecular targets and their binding energies. 5

5. Table S5. Predicted interaction of thienopyridines (4) with bio-molecular targets and their binding energies. 5

6. Table S6. Predicted interaction of thienopyridines (5) with bio-molecular targets and their binding energies. 6

7. Table S7. Predicted interaction of thienopyridines (6) with bio-molecular targets and their binding energies. 6

8. GPCR MAX Panel Primary screen, Agonist and Antagonist. 7

9. TableS8. Assay mode: Agonist, 10 µM of 1. 8

10. Table S9. Assay mode: Antagonist, 10 µM of 1 10

11. GPCR MAX dose response, Agonist and Antagonist 13

12. Table S10. Results of the dose response experiments for the receptors 15

13. Gene expression for the NCI-60 human tumour cell lines 17

14. ATOX1 18

14.1. Table S11. Average transcript intensity z scores (ATOX1) 19

15. 1-phosphatidylinositol 4,5-bisphosphate phosphodiesterase PLCδ1 21

15.1. Table S12. Average transcript intensity z scores (PLC δ1) 22

16.1-phosphatidylinositol 4,5-bisphosphate phosphodiesterase PLCδ3 24

16.1. Table S13: Average transcript intensity z scores (PLC δ3) 25

17. TDP1 27

17.1. Table S14. Average transcript intensity z scores (TDP1) 28

18. Tubulin 30

18.1. Tubulin Beta 2B 30

18.1.1. Table S15. Average transcript intensity z scores (Tubulin Beta 2B) 31

18.2. Tubulin Alpha 1A 33

18.2.1. Table S16. Average transcript intensity z scores (Tubulin Alpha 1A) 34

19. $A_{2A}AR$ 36

19.1. Table S17. Average transcript intensity z scores ($A_{2A}AR$) 37

20. CXCR4 39

20.1. Table S18. Average transcript intensity z scores (CXCR4) 40

21. GPR35 42

21.1. TableS19. Average transcript intensity z scores (GPR35) 43

22. NPSRB1 45

22.1. TableS20. Average transcript intensity z scores (NPSRB1) 46

1 Table S1. RMSD values (Å) of re-docked co-crystallised ligands.

SR. No.	Proteins	Co-Crystallised Ligand	GS	CS	PLP	ASP	RMSD(Avg.)
1	PLC-δ1	Inositol 1,4,5-triphosphate	5.8	1.4	1.1	1.3	2.4
2	Tubulin-Colchicine Complex	**LOC** N-[(7S)-1,2,3,10-tetramethoxy-9-oxo-6,7-dihydro-5H-benzo[d]heptalen-7-yl]ethanamide	0.95	0.86	0.93	1.0	0.93
3	$A_{2A}AR$	**ZMA** 4-{2-[(7-amino-2-furan-2-yl[1,2,4]triazolo[1,5- a][1,3,5]triazin-5-yl)amino]ethyl}phenol	0.82	2.7	1.7	1.8	1.7
4	CXCR4	**ITD** (6,6-dimethyl-5,6-dihydroimidazo[2,1-b][1,3]thiazol- 3-yl)methyl N,N'-dicyclohexylimidothiocarbamate	4.4	3.6	6.9	6.4	5.3
5	GPR35	Bufrolin	3.0	1.7	1.7	2.9	2.3

2 Table S2. Predicted affinities of (1) with the bio-molecular targets

GOLD						Glide
**No.**	**Targets**	**GS**	**CS**	**PLP**	**ASP**	
1	PLC-δ1	57.4	31.2	63.6	34.2	-4.7
2	ATOX1	44.4	20.8	39.9	15.1	-2.6
3	TDP1	49.9	28.2	47.6	32.5	-3.8
4	Tubulin-ColchicineComplex	63.9	31.4	54.9	25.4	-6.7
5	$A_{2A}AR$	67.2	44.1	69.0	42.2	-8.5
6	CXCR4	70.0	33.3	65.6	40.9	-6.4
7	CRL-RAMP3	56.7	33.6	61.8	36.2	-5.5
8	GPR35	65.9	33.3	63.7	37.9	-4.4
9	NSPRb1	53.4	28.2	61.1	32.9	-5.1
10	PRLHR	54.5	37.9	52.3	32.9	-6.4

3 Table S3. Predicted interaction of thienopyridines (2) with bio-molecular targets and their binding energies

		GOLD				Glide
**SR.No.**	**Targets**	**GS**	**CS**	**PLP**	**ASP**	
1	PLC-δ1	63.51	30.3	66.8	33.7	-3.1
2	ATOX1	40.3	20.3	39.1	14.9	-2.0
3	TDP1	50.4	27.7	45.9	30.4	-1.9
4	Tubulin-Colchicine Complex	62.5	29.6	61.3	28.0	-7.0
5	$A_{2A}AR$	61.0	36.9	67.4	39.8	-8.5
6	CXCR4	61.1	34.5	64.9	38.5	-6.4
**7**	CRL-RAMP3	58.3	32.6	60.5	35.7	-5.8
8	GPR35	61.1	33.3	59.1	38.2	-5.4
9	NSPRb1	50.4	28.4	55.4	31.9	-4.1
10	PRLHR	53.7	34.6	57.0	32.9	-5.9

4 Table S4. Predicted affinities of (3) with the bio-molecular targets

		GOLD				Glide
**SR.No.**	**Proteins**	**GS**	**CS**	**PLP**	**ASP**	
1	PLC-δ1	56.1	28.2	64.3	34.8	-2.4
2	ATOX1	40.1	20.9	48.5	14.4	-1.9
3	TDP1	48.1	26.7	55.9	32.9	-4.1
4	Tubulin-Colchicine Complex	63.2	25.9	53.4	27.8	-5.7
5	$A_{2A}AR$	67.4	35.6	73.1	41.2	-9.3
6	CXCR4	65.5	32.6	68.8	33.9	-6.7
7	CRL-RAMP3	57.6	30.4	72.4	40.2	-5.8
8	GPR35	64.4	32.6	69.5	39.3	-4.4
9	NSPRb1	47.7	28.9	64.8	31.0	-3.7
10	PRLHR	59.9	29.2	52.4	34.5	-4.1

5 Table S5. Predicted affinities of (4) with the bio-molecular targets

		GOLD				Glide
**SR.No.**	**Proteins**	**GS**	**CS**	**PLP**	**ASP**	
1	PLC-δ1	54.4	26.8	59.3	34.6	-4.5
2	ATOX1	40.2	21.4	40.8	15.7	-2.4
3	TDP1	48.9	27.2	47.6	28.9	-2.3
4	Tubulin-Colchicine Complex	64.4	28.9	68.1	24.8	-7.4
5	$A_{2A}AR$	65.5	38.9	68.7	37.9	-8.4
6	CXCR4	58.3	32.7	65.9	33.9	-6.1
7	CRL-RAMP3	57.8	33.4	65.1	33.9	-5.8
8	GPR35	62.5	32.6	63.0	36.9	-5.2
9	NSPRb1	48.7	27.3	51.4	29.2	-4.2
10	PRLHR	56.4	30.9	58.6	30.6	-5.2

6 Table S6. Predicted affinities of (5) with the bio-molecular targets

GOLD						Glide
**SR.No.**	**Proteins**	**GS**	**CS**	**PLP**	**ASP**	
1	PLC-δ1	59.1	28.4	63.9	36.7	-3.7
2	ATOX1	40.6	19.0	39.9	18.9	-2.3
3	TDP1	49.9	26.3	47.9	31.7	-3.8
4	Tubulin-Colchicine Complex	62.3	26.4	52.7	28.7	-7.4
5	$A_{2A}AR$	61.7	36.9	68.8	40.9	-8.9
6	CXCR4	61.8	30.6	65.8	40.1	-6.4
7	CRL-RAMP3	57.8	28.6	62.3	37.9	-5.7
8	GPR35	60.7	28.7	59.1	41.3	-5.1
9	NSPRb1	51.1	23.8	57.7	31.9	-4.5
10	PRLHR	60.6	31.6	59.1	34.4	-5.8

7 Table S7. Predicted affinities of derivative (6) with the putative bio-molecular targets

GOLD						Glide
**SR.No.**	**Proteins**	**GS**	**CS**	**PLP**	**ASP**	
1	PLC-δ1	50.9	26.5	60.4	36.6	-3.9
2	ATOX1	43.3	17.1	38.8	17.4	-1.9
3	TDP1	50.7	24.9	46.4	30.6	-3.1
4	Tubulin-Colchicine Complex	65.7	32.7	63.4	31.9	-5.8
5	$A_{2A}AR$	62.4	39.2	68.2	40.5	-9.2
6	CXCR4	59.1	33.4	67.5	36.9	-5.8
7	CRL-RAMP3	54.1	31.6	62.3	36.3	-5.6
8	GPR35	62.9	32.2	61.4	36.7	-3.7
9	NSPRb1	48.1	26.0	58.4	29.4	-4.2
10	PRLHR	54.9	32.4	61.7	32.5	-5.6

8 GPCR MAX Panel Primary screen, Agonist and Antagonist

The assays were performed utilising the PathHunter beta-arrestin enzyme fragment complementation (EFC) technology.

Cell Handling

1. PathHunter cell lines were expanded from freezer stocks according to standard procedures.

2. Cells were seeded in a total volume of 20 µL into white walled, 384-well microplates and incubated at 37°C for the appropriate time prior to testing.

Agonist Format

1. For agonist determination, cells were incubated with sample to induce response.

2. Intermediate dilution of sample stocks was performed to generate 5X sample in assay buffer.

3. 5 µL of 5x sample was added to cells and incubated at 37°C or room temperature for 90 or 180 minutes. Final assay vehicle concentration was 1%.

Antagonist Format

1. For antagonist determination, cells were pre-incubated with antagonist followed by agonist challenge at the EC_{80} concentration.

2. Intermediate dilution of sample stocks was performed to generate 5X sample in assay buffer.

3. 5 µL of 5xsample was added to cells and incubated at 37°C or room temperature for 30 minutes. Vehicle concentration was 1%.

4. 5 µL of 6X EC_{80} agonist in assay buffer was added to the cells and incubated at 37°C or room temperature for 90 or 180 minutes.

Signal Detection

1. Assay signal was generated through a single addition of 12.5 or 15 µL (50% v/v) of PathHunter

Detection reagent cocktail, followed by a one hour incubation at room temperature.

2. Microplates were read following signal generation with a PerkinElmer EnvisionTM instrument for chemiluminescent signal detection.

Data Analysis

Compound activity was analysed using CBIS data analysis suite (ChemInnovation, CA).

1. For agonist mode assays, percentage activity was calculated using the following formula:

% Activity =100% x (mean RLU of test sample — mean RLU of vehicle control) / (mean MAX control ligand — mean RLU of vehicle control).

2. For antagonist mode assays, percentage inhibition was calculated using the following formula:

% Inhibition =100% x (1— (mean RLU of test sample— mean RLU of vehicle control) / (mean RLU of EC_{80} control — mean RLU of vehicle control)).

9 **Table S8.** Assay mode: Agonist, 10 µM of 1

**GPCR ID**	**Mean RLU**	**% Activity**	**GPCR ID**	**Mean RLU**	**% Activity**
ADCYAP1R1	156300	2%	GLP2R	165400	0%
ADORA3	160400	2%	GPR1	35600	0%
ADRA1B	351400	1%	GPR103	84800	-2%
ADRA2A	403500	-1%	GPR109A	791800	5%
ADRA2B	196200	-3%	GPR109B	1053700	0%
ADRA2C	340700	-1%	GPR119	318800	-5%
ADRB1	201800	4%	GPR120	22600	1%
ADRB2	34700	0%	GPR35	1101900	55%
AGTR1	689700	5%	GPR92	207100	3%
AGTRL1	516600	5%	GRPR	44900	0%
AVPR1A	45300	2%	HCRTR1	32600	0%
AVPR1B	47000	4%	HCRTR2	55700	0%
AVPR2	689900	1%	HRH1	200100	-1%
BDKRB1	25300	-1%	HRH2	84200	4%
BDKRB2	537900	1%	HRH3	71900	-1%
BRS3	174700	1%	HRH4	558000	-5%
C3AR1	91900	1%	HTR1A	636000	3%
C5AR1	132300	0%	HTR1B	1811900	2%
C5L2	1021800	12%	HTR1E	112000	0%
CALCR	57000	0%	HTR1F	334400	4%
CALCRL-RAMP1	81000	2%	HTR2A	311800	3%
CALCRL-RAMP2	341400	6%	HTR2C	432600	1%
CALCRL-RAMP3	446400	-1%	HTR5A	553600	0%

CALCR-RAMP2	142300	5%	KISS1R	43500	-2%
CALCR-RAMP3	52300	2%	LHCGR	101700	-1%
CCKAR	20400	-1%	LTB4R	138600	0%
CCKBR	683300	-1%	MC1R	15600	1%
CCR10	95600	2%	MC3R	91400	-1%
CCR1	776500	0%	MC4R	43600	11%
CCR2	177100	3%	MC5R	122600	1%
CCR3	164400	10%	MCHR1	56400	5%
CCR4	207700	3%	MCHR2	84100	2%
CCR5	70100	1%	MLNR	220900	1%
CCR6	48600	0%	MRGPRX1	758200	1%
CCR7	557900	4%	MRGPRX2	430900	9%
CCR8	19800	0%	MTNR1A	76200	0%
CCR9	134200	1%	NMBR	83200	2%
CHRM1	198500	3%	NMU1R	117500	4%
CHRM2	42700	0%	NPBWR1	133400	3%
CHRM3	32700	1%	NPBWR2	166600	0%
CHRM4	1014300	-7%	NPFFR1	144100	-1%
CHRM5	1087900	3%	NPSR1B	46300	0%
CMKLR1	75600	0%	NPY1R	34300	0%
CNR1	73400	0%	NPY2R	189300	-1%
CNR2	285700	-17%	NTSR1	408400	-1%
CRHR1	338600	2%	OPRD1	94800	-1%
CRHR2	111100	0%	OPRK1	24000	4%
CRTH2	140400	4%	OPRL1	161100	1%
CX3CR1	299800	0%	OPRM1	123900	2%
CXCR1	304300	3%	OXER1	174800	-1%
CXCR2	121800	2%	OXTR	34800	0%
CXCR3	304000	5%	P2RY1	99200	-2%
CXCR4	144600	2%	P2RY11	63100	0%
CXCR5	785900	-1%	P2RY12	294900	13%
CXCR6	45600	1%	P2RY2	433000	5%
CXCR7	852600	20%	P2RY4	221800	11%
DRD1	53100	-1%	P2RY6	676600	17%
DRD2L	84200	-1%	PPYR1	48200	1%
DRD2S	175300	1%	PRLHR	63900	1%
DRD3	736100	-4%	PROKR1	25800	1%
DRD4	15600	-1%	PROKR2	41700	0%
DRD5	66400	8%	PTAFR	720900	0%
EBI2	124100	0%	PTGER2	27200	2%
EDG1	164600	0%	PTGER3	396100	0%
EDG3	728300	4%	PTGER4	148900	0%
EDG4	140200	-6%	PTGFR	14200	1%
EDG5	189400	0%	PTGIR	305400	3%

EDG6	1010600	4%	PTHR1	128800	0%
EDG7	78400	0%	PTHR2	143700	2%
EDNRA	32900	1%	RXFP3	89500	-2%
EDNRB	68000	1%	SCTR	575400	4%
F2R	174200	0%	SSTR1	64900	19%
F2RL1	879500	11%	SSTR2	13400	0%
F2RL3	757600	1%	SSTR3	57500	0%
FFAR1	709800	47%	SSTR5	345300	1%
FPR1	904800	1%	TACR1	465800	-1%
FPRL1	66400	0%	TACR2	401700	2%
FSHR	155300	0%	TACR3	133100	-1%
GALR1	311200	1%	TBXA2R	175300	-3%
GALR2	400000	-1%	TRHR	21400	1%
GCGR	473900	0%	TSHR(L)	13800	7%
GHSR	227500	-1%	UTR2	24900	-2%
GIPR	35100	-3%	VIPR1	263500	1%
GLP1R	103800	0%	VIPR2	341200	0%

Table S9. Assay mode: Antagonist, 10 μM of **1**

**GPCR ID**	**Mean RLU**	**% Inhibition**	**GPCR ID**	**Mean RLU**	**% Inhibition**
ADCYAP1R1	831700	23%	GLP2R	977900	16%
ADORA3	458900	25%	GPR1	696700	7%
ADRA1B	2414600	11%	GPR103	272600	-5%
ADRA2A	1272000	8%	GPR109A	1915000	-13%
ADRA2B	940600	-9%	GPR109B	4365000	7%
ADRA2C	1994900	-14%	GPR119	474000	23%
ADRB1	1234200	-27%	GPR120	99600	4%
ADRB2	971600	-46%	GPR35	1455200	-12%
AGTR1	2469500	7%	GPR92	753200	-28%
AGTRL1	2761200	5%	GRPR	1277900	11%
AVPR1A	938600	-12%	HCRTR1	2690200	13%
AVPR1B	284300	3%	HCRTR2	2727600	8%
AVPR2	2797800	13%	HRH1	1774400	13%
BDKRB1	166800	-9%	HRH2	296500	10%
BDKRB2	3633300	1%	HRH3	348100	-14%
BRS3	938900	17%	HRH4	1758800	32%
C3AR1	2503300	-3%	HTR1A	2014500	3%
C5AR1	1409400	2%	HTR1B	2492000	42%
C5L2	2384800	-20%	HTR1E	178900	19%
CALCR	442200	-18%	HTR1F	998100	-21%
CALCRL-RAMP1	869700	6%	HTR2A	1315600	-1%

CALCRL-RAMP2	1525400	-23%	HTR2C	1859500	-4%
CALCRL-RAMP3	1218100	63%	HTR5A	2641100	2%
CALCR-RAMP2	608700	0%	KISS1R	261400	5%
CALCR-RAMP3	87600	17%	LHCGR	340000	-2%
CCKAR	662900	6%	LTB4R	1876500	4%
CCKBR	2837900	12%	MC1R	60500	-16%
CCR10	1396100	-49%	MC3R	253300	2%
CCR1	2485800	-10%	MC4R	243700	-42%
CCR2	1142800	-8%	MC5R	287100	20%
CCR3	579400	-47%	MCHR1	641900	-77%
CCR4	1087800	26%	MCHR2	598000	-7%
CCR5	869200	11%	MLNR	2110300	-18%
CCR6	1002600	-6%	MRGPRX1	4295000	6%
CCR7	2063200	34%	MRGPRX2	1756000	-1%
CCR8	665600	-7%	MTNR1A	260700	-15%
CCR9	2191100	17%	NMBR	919100	28%
CHRM1	646000	0%	NMU1R	932200	2%
CHRM2	424700	-14%	NPBWR1	459500	-14%
CHRM3	244600	14%	NPBWR2	1052300	-5%
CHRM4	2312100	-10%	NPFFR1	468400	-55%
CHRM5	4325200	8%	NPSR1B	167200	59%
CMKLR1	2235200	14%	NPY1R	436000	11%
CNR1	684200	23%	NPY2R	3262700	-9%
CNR2	800700	16%	NTSR1	1746100	12%
CRHR1	3179600	20%	OPRD1	1047900	-26%
CRHR2	2337300	8%	OPRK1	212700	-162%
CRTH2	592000	13%	OPRL1	741900	-7%
CX3CR1	3271300	11%	OPRM1	1508300	-18%
CXCR1	3443800	2%	OXER1	446900	-1%
CXCR2	510500	33%	OXTR	768000	7%
CXCR3	1393200	-19%	P2RY1	402000	24%
CXCR4	212700	39%	P2RY11	493100	-6%
CXCR5	2542000	0%	P2RY12	692000	-25%
CXCR6	160300	5%	P2RY2	1541000	-14%
CXCR7	2643100	-15%	P2RY4	644300	-16%
DRD1	437600	12%	P2RY6	1598600	-17%
DRD2L	534300	-1%	PPYR1	327500	-18%
DRD2S	931300	-6%	PRLHR	260200	44%
DRD3	1734800	20%	PROKR1	392800	6%
DRD4	78000	-7%	PROKR2	881800	-3%
DRD5	507900	-31%	PTAFR	4935500	2%
EBI2	1625500	4%	PTGER2	105200	-5%
EDG1	1153200	-6%	PTGER3	1504500	11%
EDG3	3732600	-2%	PTGER4	1131300	7%

EDG4	428500	7%	PTGFR	437900	-13%
EDG5	2450900	-1%	PTGIR	863000	8%
EDG6	1525400	-6%	PTHR1	1747700	12%
EDG7	564300	-8%	PTHR2	2939600	7%
EDNRA	976200	-18%	RXFP3	256600	-27%
EDNRB	1228300	0%	SCTR	2193200	13%
F2R	1056900	5%	SSTR1	134300	-62%
F2RL1	3372600	11%	SSTR2	484800	9%
F2RL3	3124200	9%	SSTR3	604100	5%
FFAR1	1206800	-59%	SSTR5	2138300	-3%
FPR1	2762900	16%	TACR1	4436900	-3%
FPRL1	2696200	11%	TACR2	1779000	14%
FSHR	397000	30%	TACR3	1538400	5%
GALR1	1660800	12%	TBXA2R	1261300	-1%
GALR2	2493000	-5%	TRHR	303200	13%
GCGR	4324200	12%	TSHR(L)	77400	-24%
GHSR	1376300	-2%	UTR2	162900	10%
GIPR	149700	2%	VIPR1	2891100	7%
GLP1R	2305300	-1%	VIPR2	4311400	12%

11 GPCR MAX dose response, Agonist and Antagonist

Targets (60 Data points each)**:**

i) CALCRL-RAMP3-Antagonist
ii) GPR35-Agonist
iii) NPSR1B-Antagonist
iv) CXCR4 - Antagonist
v) HTR1B - Antagonist
vi) PRLHR - Antagonist

Cell Handling

1. PathHunter cell lines were expanded from freezer stocks according to standard procedures.

2. Cells were seeded in a total volume of 20 µL into white walled, 384-well microplates and incubated at 37°C for the appropriate time prior to testing.

Agonist Format

1. For agonist determination, cells were incubated with sample to induce response.

2. Intermediate dilution of sample stocks was performed to generate 5X sample in assay buffer.

3. 5 µL of 5X sample was added to cells and incubated at 37°C or room temperature for 90 to 180 minutes. Vehicle concentration was 1%.

Inverse Agonist Format

1. For inverse agonist determination, cells were incubated with sample to induce response.

2. Intermediate dilution of sample stocks was performed to generate 5X sample in assay buffer.

3. 5 µL of 5X sample was added to cells and incubated at 37°C or room temperature for 3 to 5 hours. Vehicle concentration was 1%. Extended incubation is typically required to observe an inverse agonist response in the PathHunter arrestin assay.

Positive Allosteric Modulation Format

1. For allosteric determination, cells were pre-incubated with sample followed by agonist induction at the EC20 concentration.

2. Intermediate dilution of sample stocks was performed to generate 5X sample in assay buffer.

3. 5 µL of 5x sample was added to cells and incubated at 37°C or room temperature for 30 minutes. Vehicle concentration was 1%.

4. 5 µLof 6X EC_{20} agonist in assay buffer was added to the cells and incubated at 37°C or room temperature for 90 or 180 minutes.

Antagonist/Negative Allosteric Modulation Format

1. For antagonist determination, cells were pre-incubated with antagonist followed by agonist challenge at the EC_{80} concentration.

2. Intermediate dilution of sample stocks was performed to generate 5X sample in assay buffer.

3. 5 µL of 5x sample was added to cells and incubated at 37°C or room temperature for 30 minutes. Vehicle concentration was 1%.

4. 5 µLof 6X EC80 agonist in assay buffer was added to the cells and incubated at 37°C or room temperature for 90 or 180 minutes.

Signal Detection

1. Assay signal was generated through a single addition of 12.5 or 15 µL (50% v/v) of PathHunter Detection reagent cocktail, followed by a one hour incubation at room temperature.

2. Microplates were read following signal generation with a PerkinElmer EnvisionTM instrument for chemiluminescent signal detection.

Data Analysis

1. Compound activity was analyzed using CBIS data analysis suite (ChemInnovation, CA).

2. For agonist mode assays, percentage activity was calculated using the following formula:

% Activity =100% x (mean RLU of test sample - mean RLU of vehicle control) / (mean MAX control ligand - mean RLU of vehicle control).

3. For inverse agonist mode assays, percentage activity was calculated using the following formula: % Inverse Agonist Activity =100% x (1 - (mean RLU of test sample - mean RLU of vehicle control) / (mean RLU of vehicle control)).

4. For positive allosteric mode assays, percentage modulation was calculated using the following formula:

% Modulation =100% x ((mean RLU of test sample - mean RLU of EC_{20} control) / (mean RLU of MAX control ligand - mean RLU of EC_{20} control)).

5. For antagonist and negative allosteric mode assays, percentage inhibition was calculated using the following formula:

% Inhibition =100% x (1 - (mean RLU of test sample - mean RLU of vehicle control) / (mean RLU of EC_{80} control - mean RLU of vehicle control)).

12 Table S10. Results of the dose response experiments for the receptors.

Assay Target	Result Type	RC50 (uM)	Result Graph	Conc	Raw Value	Percent Efficacy
GPR35	EC50	7.5029	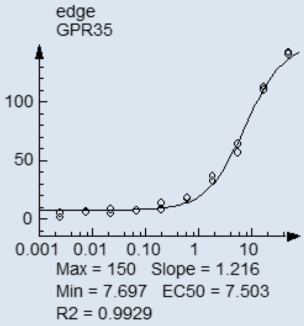	0.0025403	227400	5.1
				0.0025403	202400	1.5
				0.0076208	233200	6.0
				0.0076208	227800	5.2
				0.022862	247200	8.0
				0.022862	220800	4.2
				0.068587	240600	7.1
				0.068587	241400	7.2
				0.20576	284800	13.5
				0.20576	245600	7.8
				0.61728	315800	18.1
				0.61728	311800	17.5
				1.8519	413400	32.4
				1.8519	441400	36.4
				5.5556	629400	64.0
				5.5556	578000	56.4
				16.667	947400	110.5
				16.667	967000	113.4
				50	1155400	141.0
				50	1166400	142.6
CALCRL-RAMP3	IC50	11.905	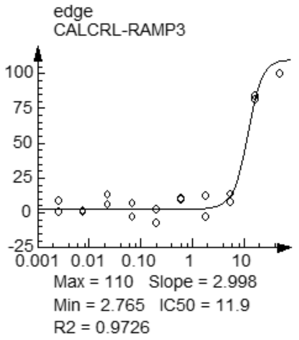	0.0025403	1811400	0.9
				0.0025403	1700800	8.4
				0.0076208	1811800	0.8
				0.0076208	1800800	1.6
				0.022862	1742000	5.6
				0.022862	1638600	12.6
				0.068587	1872200	-3.3
				0.068587	1728400	6.5
				0.20576	1929800	-7.2
				0.20576	1790400	2.3
				0.61728	1672200	10.3
				0.61728	1688000	9.2
				1.8519	1872600	-3.3
				1.8519	1650400	11.8
				5.5556	1708400	7.8
				5.5556	1626800	13.4
				16.667	586200	83.9
				16.667	621400	81.5
				50	346200	100.1
				50	348200	100.0

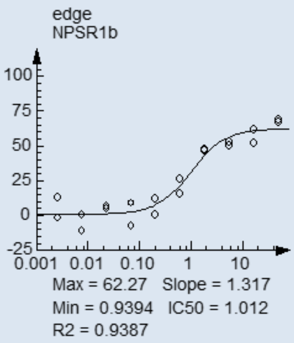

NPSR1b	IC50	1.0117	0.0025403	289000	-1.7
			0.0025403	253400	13.2
			0.0076208	311200	-11.0
			0.0076208	284200	0.3
			0.022862	268800	6.8
			0.022862	273800	4.7
			0.068587	302000	-7.2
			0.068587	263600	8.9
			0.20576	283200	0.7
			0.20576	255800	12.2
			0.61728	247800	15.6
			0.61728	221600	26.6
			1.8519	171800	47.4
			1.8519	173600	46.7
			5.5556	159400	52.6
			5.5556	164600	50.5
			16.667	138400	61.5
			16.667	161000	52.0
			50	124600	67.2
			50	120600	68.9

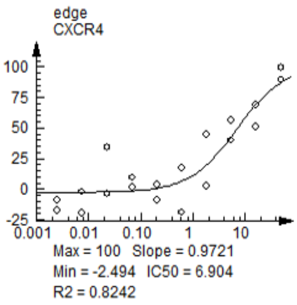

CXCR4	IC50	6.904051	0.0025403	160600	-9.174613
			0.0025403	165600	-17.39559
			0.0076208	166920	-19.56593
			0.0076208	156400	-2.268991
			0.0228624	134520	33.70602
			0.0228624	157240	-3.650115
			0.0685871	149280	9.437685
			0.0685871	154000	1.67708
			0.2057613	152880	3.518579
			0.2057613	160400	-8.845775
			0.6172839	144360	17.52713
			0.6172839	166640	-19.10556
			1.8518518	153440	2.59783
			1.8518518	128120	44.22887
			5.5555553	121000	55.93555
			5.5555553	130840	39.75666
			16.666666	123960	51.06873
			16.666666	113240	68.6945
			50	100680	89.34561
			50	94400	99.67116

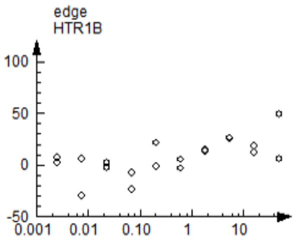

HTR1B	IC50	>50	0.0025403	2347400	6.734908
			0.0025403	2405560	1.802562
			0.0076208	2355120	6.080202
			0.0076208	2778400	-29.81669
			0.0228624	2463840	-3.139961
			0.0228624	2402320	2.077335
			0.0685871	2513280	-7.332794
			0.0685871	2701720	-23.31373
			0.2057613	2176320	21.2436
			0.2057613	2449280	-1.905178
			0.6172839	2365080	5.235529
			0.6172839	2466720	-3.384203
			1.8518518	2273600	12.99363
			1.8518518	2254840	14.5846
			5.5555553	2118560	26.14202
			5.5555553	2130320	25.1447
			16.666666	2282720	12.22019
			16.666666	2215200	17.94633
			50	2356480	5.964865
			50	1852840	48.67681

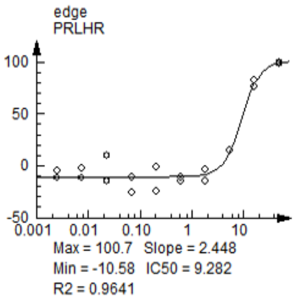

PRLHR	IC50	9.282309	0.0025403	262760	-5.056417
			0.0025403	276080	-11.66274
			0.0076208	258200	-2.794792
			0.0076208	276040	-11.6429
			0.0228624	233000	9.703658
			0.0228624	283000	-15.09485
			0.0685871	275040	-11.14693
			0.0685871	304960	-25.98636
			0.2057613	254600	-1.009299
			0.2057613	301560	-24.30006
			0.6172839	283480	-15.33292
			0.6172839	275640	-11.44451
			1.8518518	282280	-14.73776
			1.8518518	259440	-3.409796
			5.5555553	223560	14.38562
			5.5555553	224000	14.16739
			16.666666	100000	75.6677
			16.666666	86120	82.55177
			50	53720	98.6212
			50	52200	99.37508

13 Gene expression for the NCI-60 human tumour cell lines

The NCI-60 panel of human cancer cell-lines is derived from nine different tissues of origin. It was initially developed for anti-cancer drug efficacy screening by the Developmental Therapeutics Program (DTP) of the US National Cancer Institute. In addition, theNCI-60 has been extensively characterized in biological, molecular and pharmacological studies. Here we examined the expression profiles of proteins in the NCI-60. With the availability of Cell Miner (http://discover.nci.nih.gov/cellminer/), a web application for rapid retrieval of genetic and pharmacological data from the NCI-60[1, 2], this task is made feasible and relatively easy. We demonstrate how integration of bioinformatics and biological investigation lead to the identification of potent bimolecular target of anticancer thienopyridines.

References

1. W. C. Reinhold, M. Sunshine, H. Liu, S. Varma, K. W. Kohn, J. Morris, J. Doroshow and Y. Pommier, Cancer research, 2012, **72**, 3499-3511.
2. O. D. Abaan, E. C. Polley, S. R. Davis, Y. J. Zhu, S. Bilke, R. L. Walker, M. Pineda, Y. Gindin, Y. Jiang and W. C. Reinhold, Cancer research, 2013, **73**, 4372-4382.

14 ATOX1

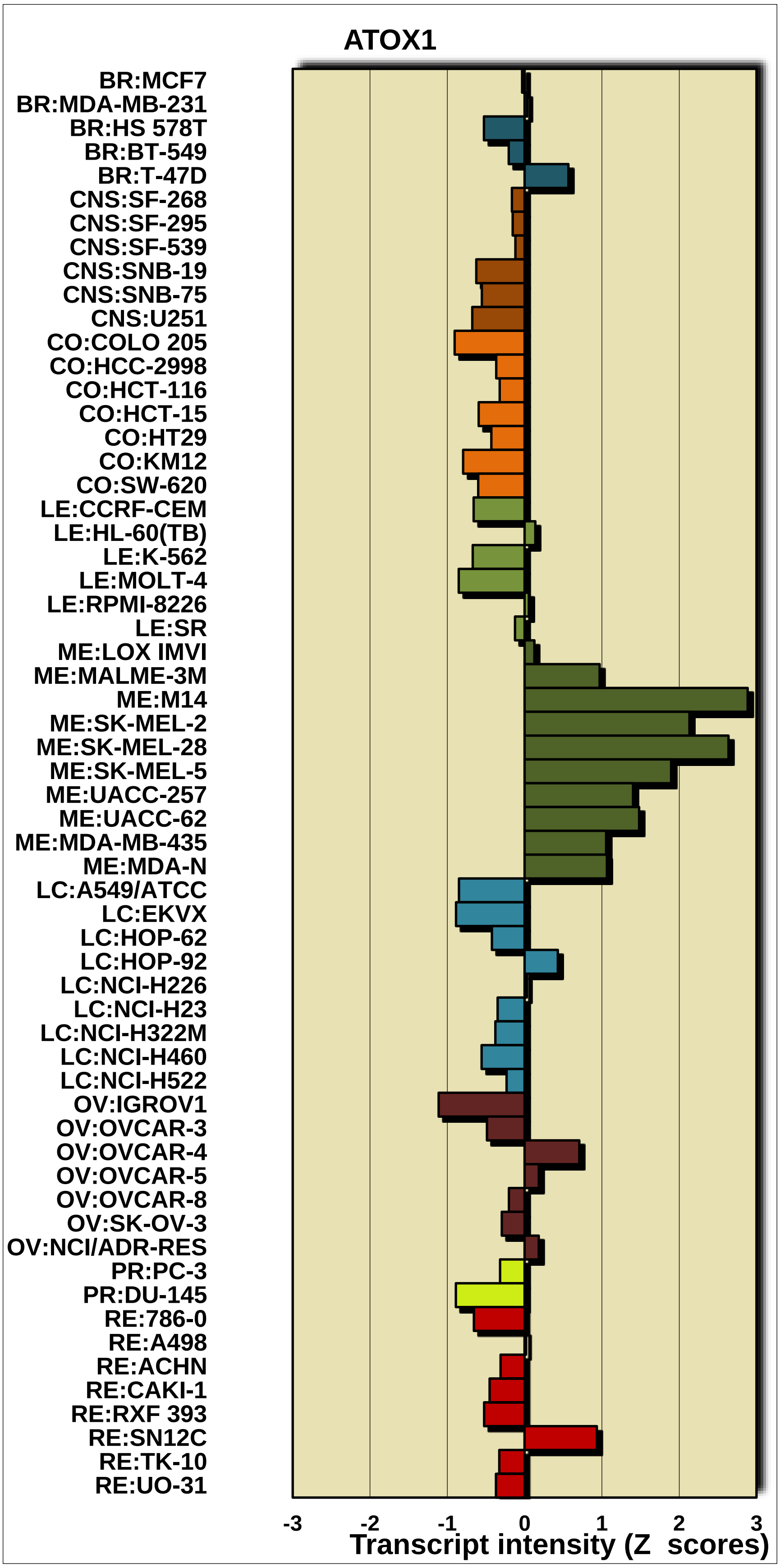

ATOX1
BR:MCF7
BR:MDA-MB-231
BR:HS 578T
BR:BT-549
BR:T-47D
CNS:SF-268
CNS:SF-295
CNS:SF-539
CNS:SNB-19
CNS:SNB-75
CNS:U251
CO:COLO 205
CO:HCC-2998
CO:HCT-116
CO:HCT-15
CO:HT29
CO:KM12
CO:SW-620
LE:CCRF-CEM
LE:HL-60(TB)
LE:K-562
LE:MOLT-4
LE:RPMI-8226
LE:SR
ME:LOX IMVI
ME:MALME-3M
ME:M14
ME:SK-MEL-2
ME:SK-MEL-28
ME:SK-MEL-5
ME:UACC-257
ME:UACC-62
ME:MDA-MB-435
ME:MDA-N
LC:A549/ATCC
LC:EKVX
LC:HOP-62
LC:HOP-92
LC:NCI-H226
LC:NCI-H23
LC:NCI-H322M
LC:NCI-H460
LC:NCI-H522
OV:IGROV1
OV:OVCAR-3
OV:OVCAR-4
OV:OVCAR-5
OV:OVCAR-8
OV:SK-OV-3
OV:NCI/ADR-RES
PR:PC-3
PR:DU-145
RE:786-0
RE:A498
RE:ACHN
RE:CAKI-1
RE:RXF 393
RE:SN12C
RE:TK-10
RE:UO-31
-3
-2
-1
0
1
2
3
Transcript intensity (Z scores)

14.1 **Table S11:** Average transcript intensity z scores (ATOX1) and GI_{50} for **1**

**Cell lines** ^{ATOX1}	**Z scores**	Average GI_{50} (nM)
**BR:MCF7**	-0.032	46.35
**BR:MDA-MB-231**	0.034	141.00
**BR:HS 578T**	-0.526	48.20
**BR:BT-549**	-0.205	320.70
**BR:T-47D**	0.567	55.00
**CNS:SF-268**	-0.164	238.00
**CNS:SF-295**	-0.154	36.50
**CNS:SF-539**	-0.119	38.20
**CNS:SNB-19**	-0.625	70.10
**CNS:SNB-75**	-0.553	40.30
**CNS:U251**	-0.677	96.20
**CO:COLO 205**	-0.905	51.80
**CO:HCC-2998**	-0.367	213.00
**CO:HCT-116**	-0.322	66.80
**CO:HCT-15**	-0.594	70.50
**CO:HT29**	-0.43	49.00
**CO:KM12**	-0.795	67.30
**CO:SW-620**	-0.6	48.60
**LE:CCRF-CEM**	-0.659	141.10
**LE:HL-60(TB)**	0.139	103.10
**LE:K-562**	-0.671	37.50
**LE:MOLT-4**	-0.852	475.50
**LE:RPMI-8226**	0.055	255.50
**LE:SR**	-0.124	74.20
**ME:LOX IMVI**	0.127	80.30
**ME:MALME-3M**	0.971	36.30
**ME:M14**	2.886	71.30
**ME:SK-MEL-2**	2.133	47.70
**ME:SK-MEL-28**	2.638	223.90
**ME:SK-MEL-5**	1.896	237.40
**ME:UACC-257**	1.403	0.00
**ME:UACC-62**	1.482	50.30
**ME:MDA-MB-435**	1.055	18.70
**ME:MDA-N**	1.065	0.00
**LC:A549/ATCC**	-0.849	63.10
**LC:EKVX**	-0.887	0.00
**LC:HOP-62**	-0.423	110.40

**LC:HOP-92**	0.43	47.90
**LC:NCI-H226**	0.028	102.40
**LC:NCI-H23**	-0.349	169.10
**LC:NCI-H322M**	-0.378	305.60
**LC:NCI-H460**	-0.556	40.80
**LC:NCI-H522**	-0.233	22.90
**OV:IGROV1**	-1.112	196.30
**OV:OVCAR-3**	-0.487	48.90
**OV:OVCAR-4**	0.708	604.50
**OV:OVCAR-5**	0.184	307.50
**OV:OVCAR-8**	-0.203	279.00
**OV:SK-OV-3**	-0.295	60.60
**OV:NCI/ADR-RES**	0.183	46.30
**PR:PC-3**	-0.317	53.50
**PR:DU-145**	-0.889	151.90
**RE:786-0**	-0.656	103.30
**RE:A498**	0.018	23.20
**RE:ACHN**	-0.31	83.70
**RE:CAKI-1**	-0.452	46.10
**RE:RXF 393**	-0.523	41.80
**RE:SN12C**	0.935	142.00
**RE:TK-10**	-0.328	0.00
**RE:UO-31**	-0.371	73.10

15 1-phosphatidylinositol 4,5-bisphosphate phosphodiesterase PLCδ1

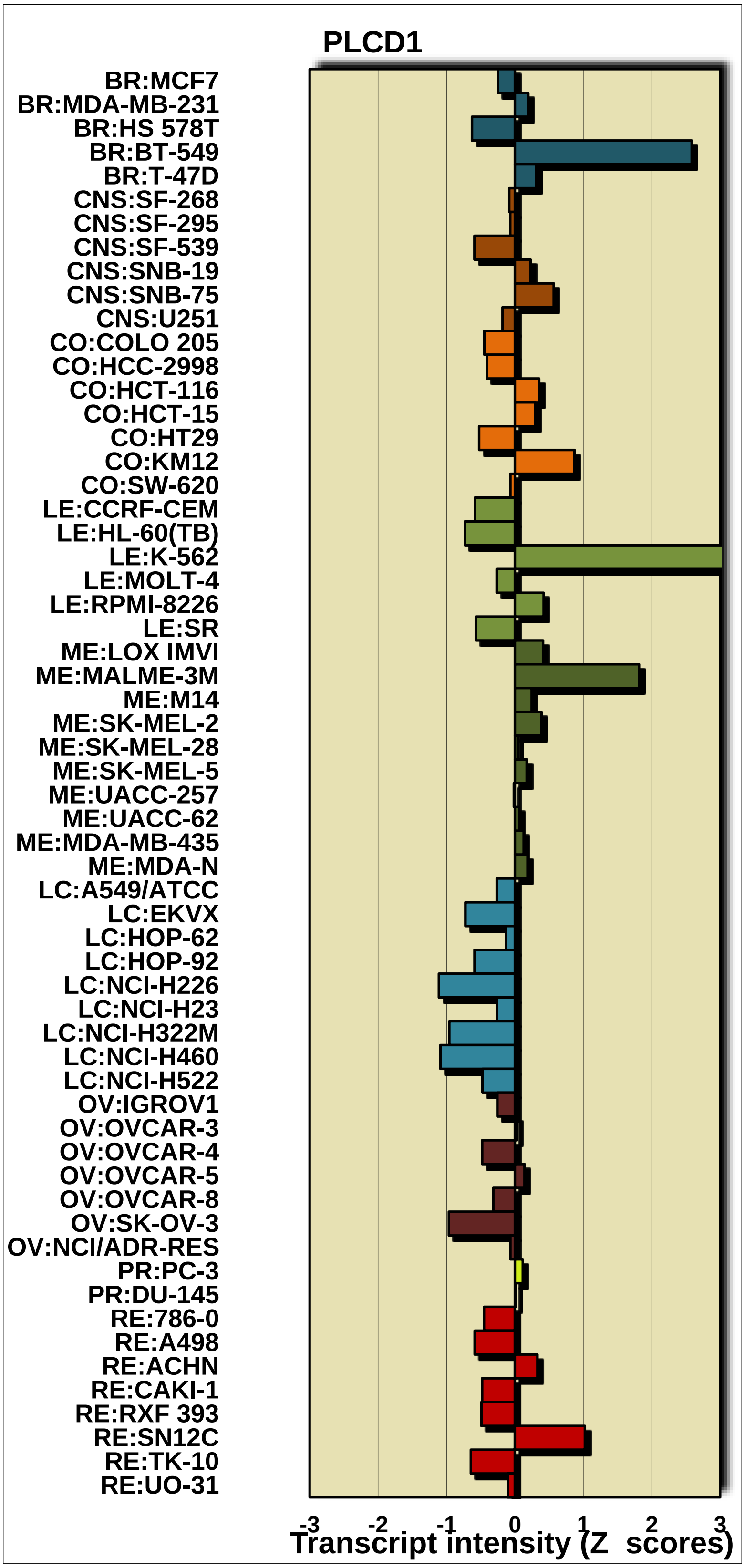

15.1 **Table S12:** Average transcript intensity z scores (PLC δ1) and GI_{50} for **1**

**Cell lines** $^{PLC\Delta 1}$	**Z scores**	Average GI_{50} (nM)
**BR:MCF7**	-0.246	46.35
**BR:MDA-MB-231**	0.196	141.00
**BR:HS 578T**	-0.627	48.20
**BR:BT-549**	2.585	320.70
**BR:T-47D**	0.311	55.00
**CNS:SF-268**	-0.083	238.00
**CNS:SF-295**	-0.068	36.50
**CNS:SF-539**	-0.591	38.20
**CNS:SNB-19**	0.229	70.10
**CNS:SNB-75**	0.568	40.30
**CNS:U251**	-0.18	96.20
**CO:COLO 205**	-0.446	51.80
**CO:HCC-2998**	-0.41	213.00
**CO:HCT-116**	0.355	66.80
**CO:HCT-15**	0.298	70.50
**CO:HT29**	-0.523	49.00
**CO:KM12**	0.872	67.30
**CO:SW-620**	-0.066	48.60
**LE:CCRF-CEM**	-0.583	141.10
**LE:HL-60(TB)**	-0.729	103.10
**LE:K-562**	4.413	37.50
**LE:MOLT-4**	-0.266	475.50
**LE:RPMI-8226**	0.421	255.50
**LE:SR**	-0.571	74.20
**ME:LOX IMVI**	0.412	80.30
**ME:MALME-3M**	1.815	36.30
**ME:M14**	0.246	71.30
**ME:SK-MEL-2**	0.389	47.70
**ME:SK-MEL-28**	0.041	223.90
**ME:SK-MEL-5**	0.173	237.40
**ME:UACC-257**	-0.015	0.00
**ME:UACC-62**	0.063	50.30
**ME:MDA-MB-435**	0.129	18.70
**ME:MDA-N**	0.185	0.00
**LC:A549/ATCC**	-0.265	63.10
**LC:EKVX**	-0.723	0.00
**LC:HOP-62**	-0.129	110.40

**LC:HOP-92**	-0.59	47.90
**LC:NCI-H226**	-1.111	102.40
**LC:NCI-H23**	-0.264	169.10
**LC:NCI-H322M**	-0.959	305.60
**LC:NCI-H460**	-1.088	40.80
**LC:NCI-H522**	-0.474	22.90
**OV:IGROV1**	-0.256	196.30
**OV:OVCAR-3**	0.031	48.90
**OV:OVCAR-4**	-0.478	604.50
**OV:OVCAR-5**	0.141	307.50
**OV:OVCAR-8**	-0.316	279.00
**OV:SK-OV-3**	-0.965	60.60
**OV:NCI/ADR-RES**	-0.066	46.30
**PR:PC-3**	0.116	53.50
**PR:DU-145**	0.014	151.90
**RE:786-0**	-0.451	103.30
**RE:A498**	-0.588	23.20
**RE:ACHN**	0.329	83.70
**RE:CAKI-1**	-0.479	46.10
**RE:RXF 393**	-0.49	41.80
**RE:SN12C**	1.023	142.00
**RE:TK-10**	-0.643	0.00
**RE:UO-31**	-0.104	73.10

16 PLCδ3

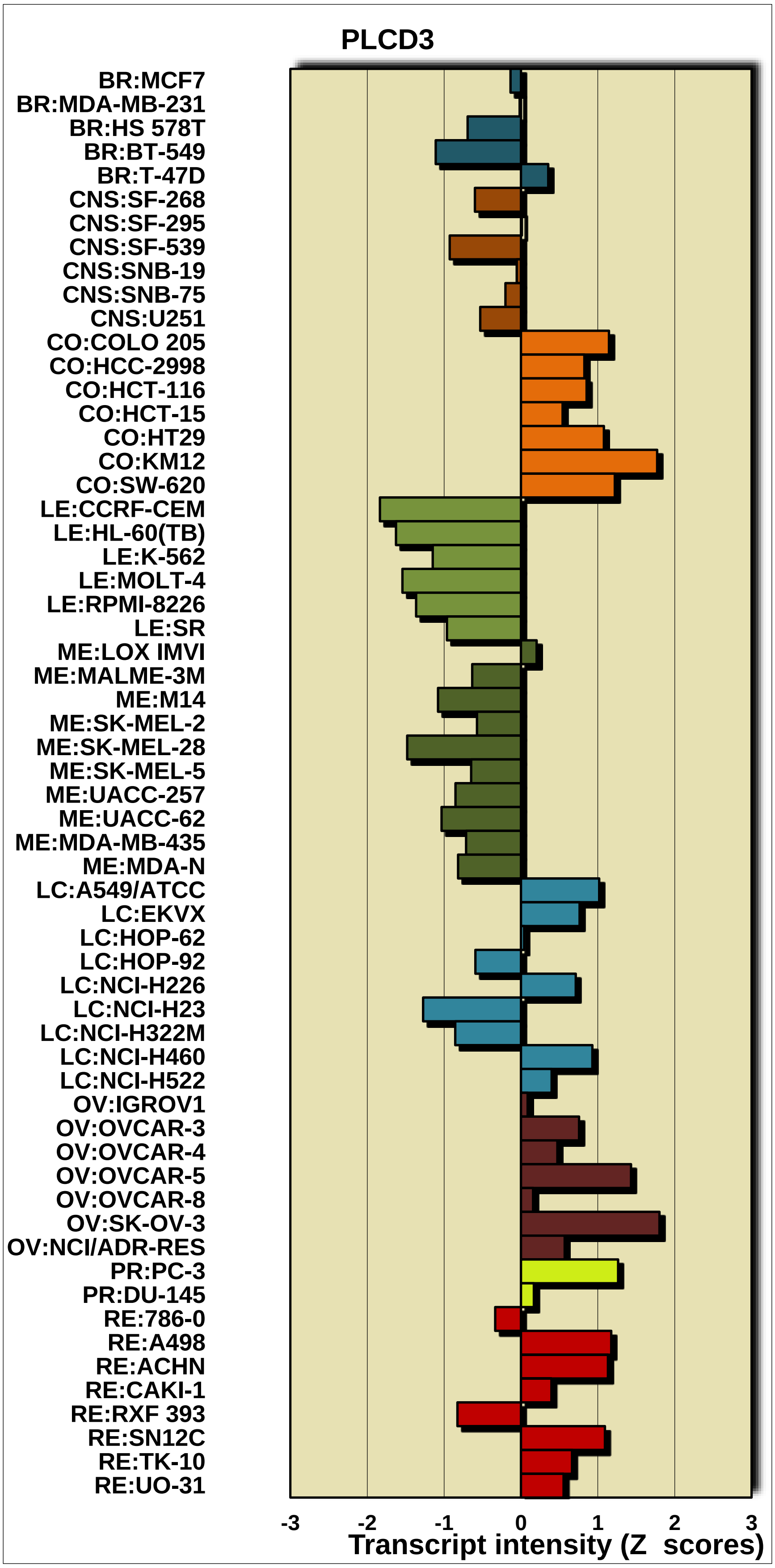
PLCD3
BR:MCF7
BR:MDA-MB-231
BR:HS 578T
BR:BT-549
BR:T-47D
CNS:SF-268
CNS:SF-295
CNS:SF-539
CNS:SNB-19
CNS:SNB-75
CNS:U251
CO:COLO 205
CO:HCC-2998
CO:HCT-116
CO:HCT-15
CO:HT29
CO:KM12
CO:SW-620
LE:CCRF-CEM
LE:HL-60(TB)
LE:K-562
LE:MOLT-4
LE:RPMI-8226
LE:SR
ME:LOX IMVI
ME:MALME-3M
ME:M14
ME:SK-MEL-2
ME:SK-MEL-28
ME:SK-MEL-5
ME:UACC-257
ME:UACC-62
ME:MDA-MB-435
ME:MDA-N
LC:A549/ATCC
LC:EKVX
LC:HOP-62
LC:HOP-92
LC:NCI-H226
LC:NCI-H23
LC:NCI-H322M
LC:NCI-H460
LC:NCI-H522
OV:IGROV1
OV:OVCAR-3
OV:OVCAR-4
OV:OVCAR-5
OV:OVCAR-8
OV:SK-OV-3
OV:NCI/ADR-RES
PR:PC-3
PR:DU-145
RE:786-0
RE:A498
RE:ACHN
RE:CAKI-1
RE:RXF 393
RE:SN12C
RE:TK-10
RE:UO-31
-3
-2
-1
0
1
2
3
Transcript intensity (Z scores)

16.1 Table S13: Average transcript intensity z scores (PLC δ3) and GI_{50} for **1**

**Cell lines** $^{PLC\delta3}$	**Z scores**	Average GI_{50} (nM)
**BR:MCF7**	-0.136	46.35
**BR:MDA-MB-231**	-0.017	141.00
**BR:HS 578T**	-0.694	48.20
**BR:BT-549**	-1.109	320.70
**BR:T-47D**	0.355	55.00
**CNS:SF-268**	-0.599	238.00
**CNS:SF-295**	0.012	36.50
**CNS:SF-539**	-0.927	38.20
**CNS:SNB-19**	-0.055	70.10
**CNS:SNB-75**	-0.202	40.30
**CNS:U251**	-0.53	96.20
**CO:COLO 205**	1.145	51.80
**CO:HCC-2998**	0.825	213.00
**CO:HCT-116**	0.854	66.80
**CO:HCT-15**	0.542	70.50
**CO:HT29**	1.079	49.00
**CO:KM12**	1.772	67.30
**CO:SW-620**	1.221	48.60
**LE:CCRF-CEM**	-1.835	141.10
**LE:HL-60(TB)**	-1.626	103.10
**LE:K-562**	-1.148	37.50
**LE:MOLT-4**	-1.542	475.50
**LE:RPMI-8226**	-1.364	255.50
**LE:SR**	-0.963	74.20
**ME:LOX IMVI**	0.204	80.30
**ME:MALME-3M**	-0.634	36.30
**ME:M14**	-1.079	71.30
**ME:SK-MEL-2**	-0.572	47.70
**ME:SK-MEL-28**	-1.48	223.90
**ME:SK-MEL-5**	-0.648	237.40
**ME:UACC-257**	-0.852	0.00
**ME:UACC-62**	-1.033	50.30
**ME:MDA-MB-435**	-0.714	18.70
**ME:MDA-N**	-0.818	0.00
**LC:A549/ATCC**	1.018	63.10
**LC:EKVX**	0.763	0.00
**LC:HOP-62**	0.042	110.40

**LC:HOP-92**	-0.593	47.90
**LC:NCI-H226**	0.713	102.40
**LC:NCI-H23**	-1.273	169.10
**LC:NCI-H322M**	-0.855	305.60
**LC:NCI-H460**	0.929	40.80
**LC:NCI-H522**	0.399	22.90
**OV:IGROV1**	0.087	196.30
**OV:OVCAR-3**	0.756	48.90
**OV:OVCAR-4**	0.474	604.50
**OV:OVCAR-5**	1.432	307.50
**OV:OVCAR-8**	0.158	279.00
**OV:SK-OV-3**	1.802	60.60
**OV:NCI/ADR-RES**	0.569	46.30
**PR:PC-3**	1.264	53.50
**PR:DU-145**	0.168	151.90
**RE:786-0**	-0.336	103.30
**RE:A498**	1.174	23.20
**RE:ACHN**	1.132	83.70
**RE:CAKI-1**	0.397	46.10
**RE:RXF 393**	-0.826	41.80
**RE:SN12C**	1.092	142.00
**RE:TK-10**	0.664	0.00
**RE:UO-31**	0.554	73.10

17 TDP1

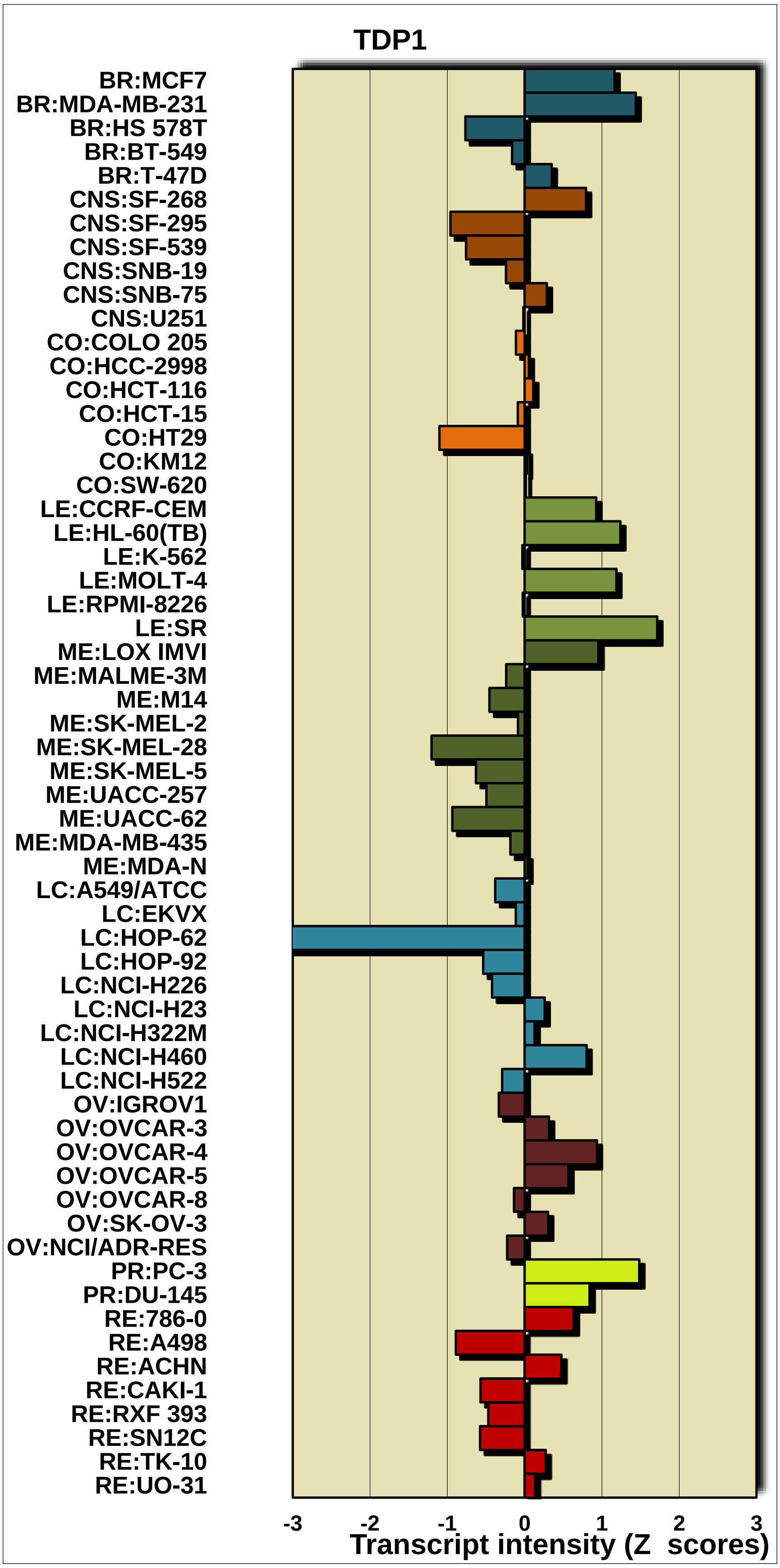

TDP1
BR:MCF7
BR:MDA-MB-231
BR:HS 578T
BR:BT-549
BR:T-47D
CNS:SF-268
CNS:SF-295
CNS:SF-539
CNS:SNB-19
CNS:SNB-75
CNS:U251
CO:COLO 205
CO:HCC-2998
CO:HCT-116
CO:HCT-15
CO:HT29
CO:KM12
CO:SW-620
LE:CCRF-CEM
LE:HL-60(TB)
LE:K-562
LE:MOLT-4
LE:RPMI-8226
LE:SR
ME:LOX IMVI
ME:MALME-3M
ME:M14
ME:SK-MEL-2
ME:SK-MEL-28
ME:SK-MEL-5
ME:UACC-257
ME:UACC-62
ME:MDA-MB-435
ME:MDA-N
LC:A549/ATCC
LC:EKVX
LC:HOP-62
LC:HOP-92
LC:NCI-H226
LC:NCI-H23
LC:NCI-H322M
LC:NCI-H460
LC:NCI-H522
OV:IGROV1
OV:OVCAR-3
OV:OVCAR-4
OV:OVCAR-5
OV:OVCAR-8
OV:SK-OV-3
OV:NCI/ADR-RES
PR:PC-3
PR:DU-145
RE:786-0
RE:A498
RE:ACHN
RE:CAKI-1
RE:RXF 393
RE:SN12C
RE:TK-10
RE:UO-31
-3 -2 -1 0 1 2 3
Transcript intensity (Z scores)

17.1 Table S14: Average transcript intensity z scores (TDP1) and GI_{50} for **1**

**Cell lines** **TDP1**	**Z scores**	Average GI_{50} (nM)
**BR:MCF7**	1.164	46.35
**BR:MDA-MB-231**	1.439	141.00
**BR:HS 578T**	-0.766	48.20
**BR:BT-549**	-0.162	320.70
**BR:T-47D**	0.351	55.00
**CNS:SF-268**	0.795	238.00
**CNS:SF-295**	-0.959	36.50
**CNS:SF-539**	-0.758	38.20
**CNS:SNB-19**	-0.241	70.10
**CNS:SNB-75**	0.288	40.30
**CNS:U251**	-0.016	96.20
**CO:COLO 205**	-0.112	51.80
**CO:HCC-2998**	0.056	213.00
**CO:HCT-116**	0.112	66.80
**CO:HCT-15**	-0.088	70.50
**CO:HT29**	-1.102	49.00
**CO:KM12**	0.03	67.30
**CO:SW-620**	0.016	48.60
**LE:CCRF-CEM**	0.927	141.10
**LE:HL-60(TB)**	1.239	103.10
**LE:K-562**	-0.029	37.50
**LE:MOLT-4**	1.188	475.50
**LE:RPMI-8226**	-0.024	255.50
**LE:SR**	1.716	74.20
**ME:LOX IMVI**	0.955	80.30
**ME:MALME-3M**	-0.238	36.30
**ME:M14**	-0.455	71.30
**ME:SK-MEL-2**	-0.086	47.70
**ME:SK-MEL-28**	-1.204	223.90
**ME:SK-MEL-5**	-0.63	237.40
**ME:UACC-257**	-0.494	0.00
**ME:UACC-62**	-0.935	50.30
**ME:MDA-MB-435**	-0.184	18.70
**ME:MDA-N**	0.039	0.00
**LC:A549/ATCC**	-0.38	63.10
**LC:EKVX**	-0.114	0.00

**LC:HOP-62**	-4.662	110.40
**LC:HOP-92**	-0.536	47.90
**LC:NCI-H226**	-0.421	102.40
**LC:NCI-H23**	0.26	169.10
**LC:NCI-H322M**	0.128	305.60
**LC:NCI-H460**	0.802	40.80
**LC:NCI-H522**	-0.29	22.90
**OV:IGROV1**	-0.334	196.30
**OV:OVCAR-3**	0.315	48.90
**OV:OVCAR-4**	0.937	604.50
**OV:OVCAR-5**	0.566	307.50
**OV:OVCAR-8**	-0.137	279.00
**OV:SK-OV-3**	0.304	60.60
**OV:NCI/ADR-RES**	-0.226	46.30
**PR:PC-3**	1.483	53.50
**PR:DU-145**	0.84	151.90
**RE:786-0**	0.635	103.30
**RE:A498**	-0.889	23.20
**RE:ACHN**	0.475	83.70
**RE:CAKI-1**	-0.571	46.10
**RE:RXF 393**	-0.469	41.80
**RE:SN12C**	-0.575	142.00
**RE:TK-10**	0.273	0.00
**RE:UO-31**	0.135	73.10

18 Tubulin

18.1 Tubulin Beta 2B

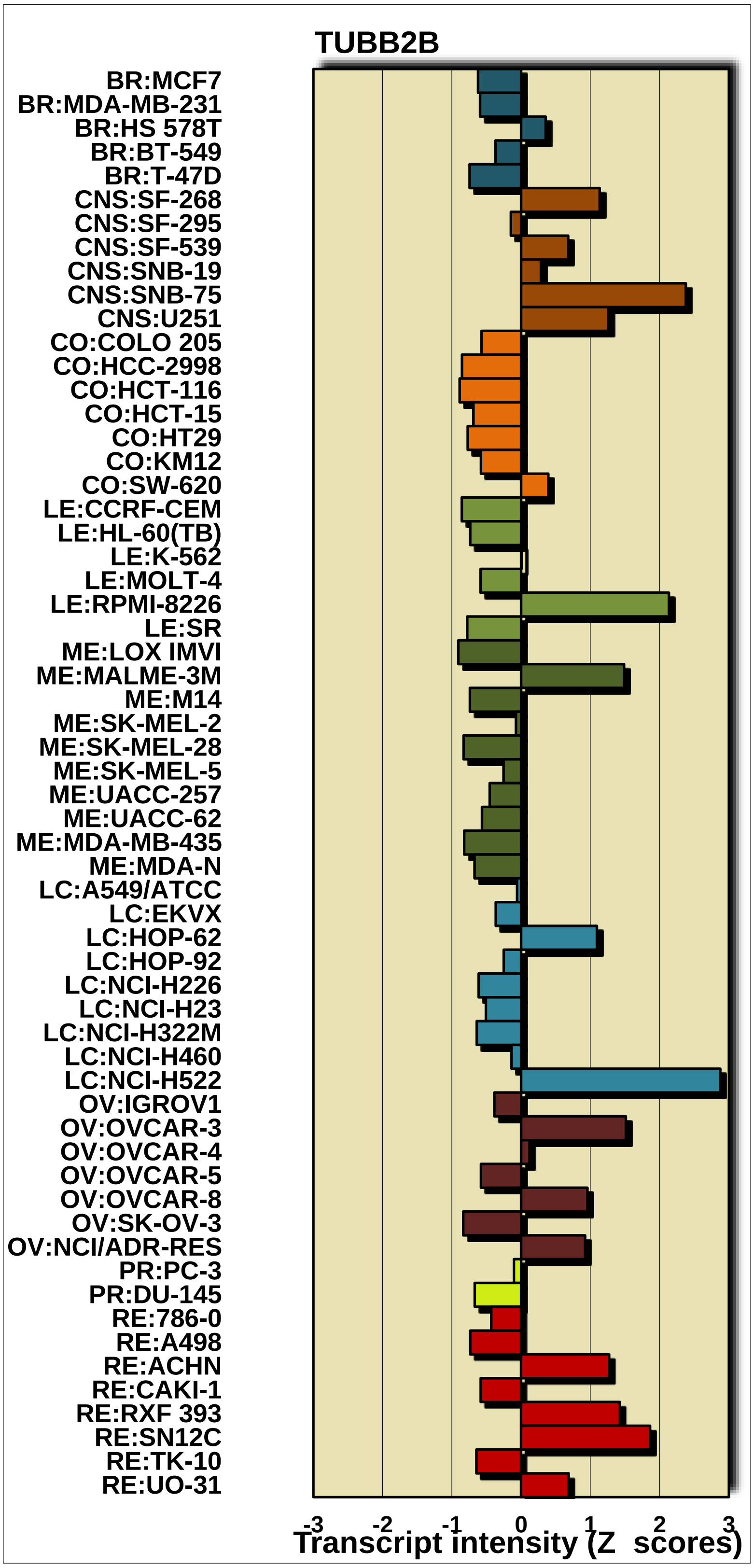

Table S15: Average transcript intensity z scores (Tubulin Beta 2B) and GI_{50} for **1**

**Cell lines** **TubulinBeta2B**	**Z scores**	Average GI_{50} (nM)
**BR:MCF7**	-0.622	46.35
**BR:MDA-MB-231**	-0.593	141.00
**BR:HS 578T**	0.355	48.20
**BR:BT-549**	-0.37	320.70
**BR:T-47D**	-0.744	55.00
**CNS:SF-268**	1.135	238.00
**CNS:SF-295**	-0.148	36.50
**CNS:SF-539**	0.68	38.20
**CNS:SNB-19**	0.286	70.10
**CNS:SNB-75**	2.379	40.30
**CNS:U251**	1.26	96.20
**CO:COLO 205**	-0.572	51.80
**CO:HCC-2998**	-0.854	213.00
**CO:HCT-116**	-0.888	66.80
**CO:HCT-15**	-0.689	70.50
**CO:HT29**	-0.771	49.00
**CO:KM12**	-0.58	67.30
**CO:SW-620**	0.393	48.60
**LE:CCRF-CEM**	-0.857	141.10
**LE:HL-60(TB)**	-0.735	103.10
**LE:K-562**	0.006	37.50
**LE:MOLT-4**	-0.585	475.50
**LE:RPMI-8226**	2.135	255.50
**LE:SR**	-0.778	74.20
**ME:LOX IMVI**	-0.907	80.30
**ME:MALME-3M**	1.487	36.30
**ME:M14**	-0.74	71.30
**ME:SK-MEL-2**	-0.075	47.70
**ME:SK-MEL-28**	-0.831	223.90
**ME:SK-MEL-5**	-0.254	237.40
**ME:UACC-257**	-0.454	0.00
**ME:UACC-62**	-0.565	50.30
**ME:MDA-MB-435**	-0.822	18.70
**ME:MDA-N**	-0.673	0.00
**LC:A549/ATCC**	-0.058	63.10
**LC:EKVX**	-0.366	0.00
**LC:HOP-62**	1.095	110.40

**LC:HOP-92**	-0.251	47.90
**LC:NCI-H226**	-0.613	102.40
**LC:NCI-H23**	-0.509	169.10
**LC:NCI-H322M**	-0.641	305.60
**LC:NCI-H460**	-0.138	40.80
**LC:NCI-H522**	2.877	22.90
**OV:IGROV1**	-0.387	196.30
**OV:OVCAR-3**	1.513	48.90
**OV:OVCAR-4**	0.122	604.50
**OV:OVCAR-5**	-0.58	307.50
**OV:OVCAR-8**	0.958	279.00
**OV:SK-OV-3**	-0.836	60.60
**OV:NCI/ADR-RES**	0.924	46.30
**PR:PC-3**	-0.104	53.50
**PR:DU-145**	-0.67	151.90
**RE:786-0**	-0.431	103.30
**RE:A498**	-0.734	23.20
**RE:ACHN**	1.271	83.70
**RE:CAKI-1**	-0.584	46.10
**RE:RXF 393**	1.426	41.80
**RE:SN12C**	1.862	142.00
**RE:TK-10**	-0.646	0.00
**RE:UO-31**	0.687	73.10

18.2 Tubulin Alpha 1A

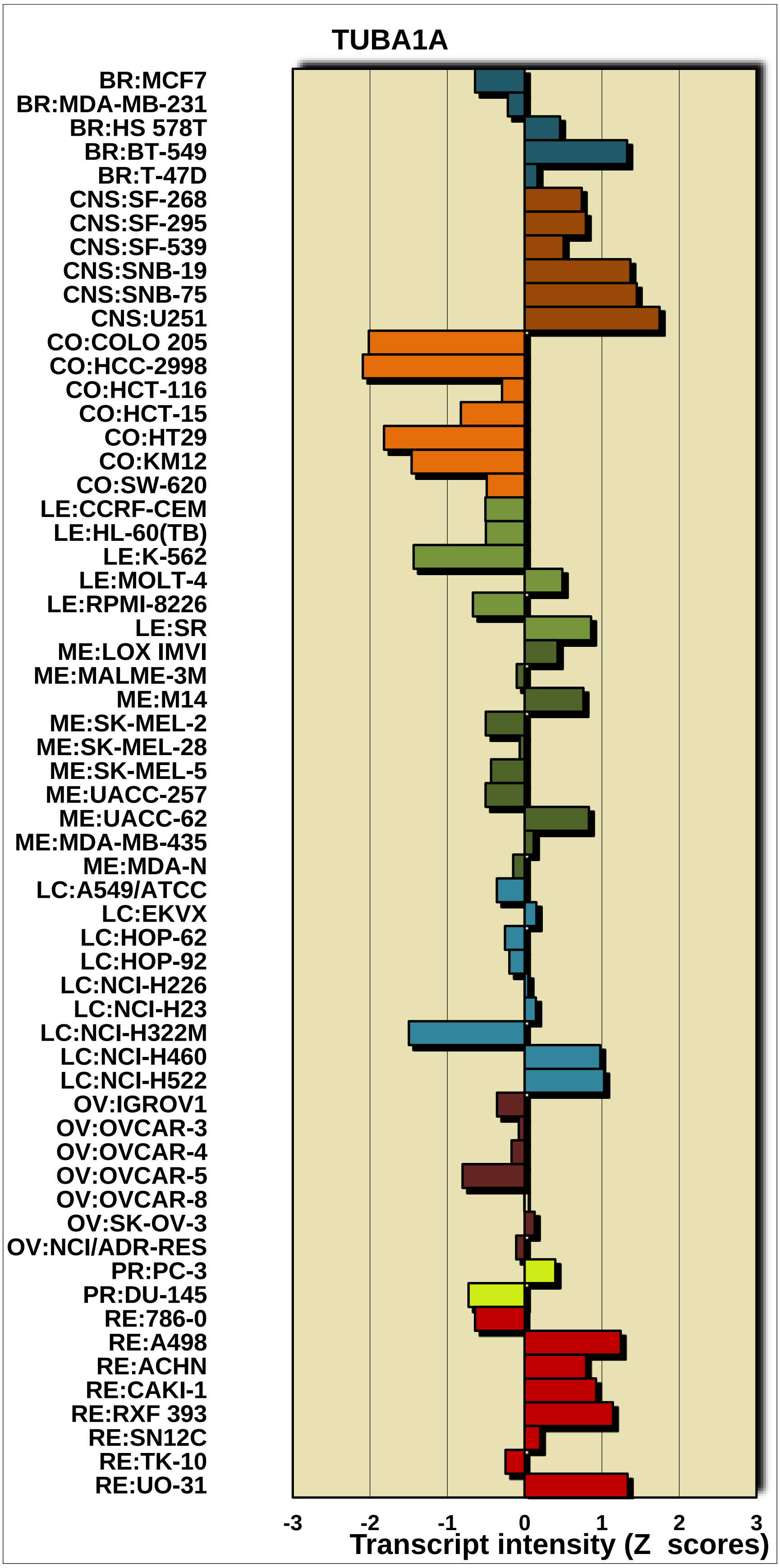

Table S16: Average transcript intensity z scores (Tubulin Alpha 1A) and GI_{50} for **1**

**Cell lines** TubulinAlpha1A	**Z scores**	Average GI_{50} (nM)
**BR:MCF7**	-0.641	46.35
**BR:MDA-MB-231**	-0.217	141.00
**BR:HS 578T**	0.459	48.20
**BR:BT-549**	1.326	320.70
**BR:T-47D**	0.166	55.00
**CNS:SF-268**	0.739	238.00
**CNS:SF-295**	0.792	36.50
**CNS:SF-539**	0.504	38.20
**CNS:SNB-19**	1.369	70.10
**CNS:SNB-75**	1.451	40.30
**CNS:U251**	1.746	96.20
**CO:COLO 205**	-2.016	51.80
**CO:HCC-2998**	-2.093	213.00
**CO:HCT-116**	-0.292	66.80
**CO:HCT-15**	-0.825	70.50
**CO:HT29**	-1.819	49.00
**CO:KM12**	-1.462	67.30
**CO:SW-620**	-0.49	48.60
**LE:CCRF-CEM**	-0.507	141.10
**LE:HL-60(TB)**	-0.501	103.10
**LE:K-562**	-1.436	37.50
**LE:MOLT-4**	0.489	475.50
**LE:RPMI-8226**	-0.669	255.50
**LE:SR**	0.86	74.20
**ME:LOX IMVI**	0.426	80.30
**ME:MALME-3M**	-0.102	36.30
**ME:M14**	0.761	71.30
**ME:SK-MEL-2**	-0.503	47.70
**ME:SK-MEL-28**	-0.064	223.90
**ME:SK-MEL-5**	-0.434	237.40
**ME:UACC-257**	-0.505	0.00
**ME:UACC-62**	0.832	50.30
**ME:MDA-MB-435**	0.117	18.70
**ME:MDA-N**	-0.148	0.00
**LC:A549/ATCC**	-0.359	63.10
**LC:EKVX**	0.152	0.00
**LC:HOP-62**	-0.254	110.40

**LC:HOP-92**	-0.197	47.90
**LC:NCI-H226**	0.048	102.40
**LC:NCI-H23**	0.146	169.10
**LC:NCI-H322M**	-1.497	305.60
**LC:NCI-H460**	0.979	40.80
**LC:NCI-H522**	1.027	22.90
**OV:IGROV1**	-0.357	196.30
**OV:OVCAR-3**	-0.076	48.90
**OV:OVCAR-4**	-0.169	604.50
**OV:OVCAR-5**	-0.803	307.50
**OV:OVCAR-8**	-0.003	279.00
**OV:SK-OV-3**	0.13	60.60
**OV:NCI/ADR-RES**	-0.109	46.30
**PR:PC-3**	0.397	53.50
**PR:DU-145**	-0.725	151.90
**RE:786-0**	-0.642	103.30
**RE:A498**	1.243	23.20
**RE:ACHN**	0.793	83.70
**RE:CAKI-1**	0.923	46.10
**RE:RXF 393**	1.142	41.80
**RE:SN12C**	0.199	142.00
**RE:TK-10**	-0.247	0.00
**RE:UO-31**	1.332	73.10

19 $A_{2A}AR$

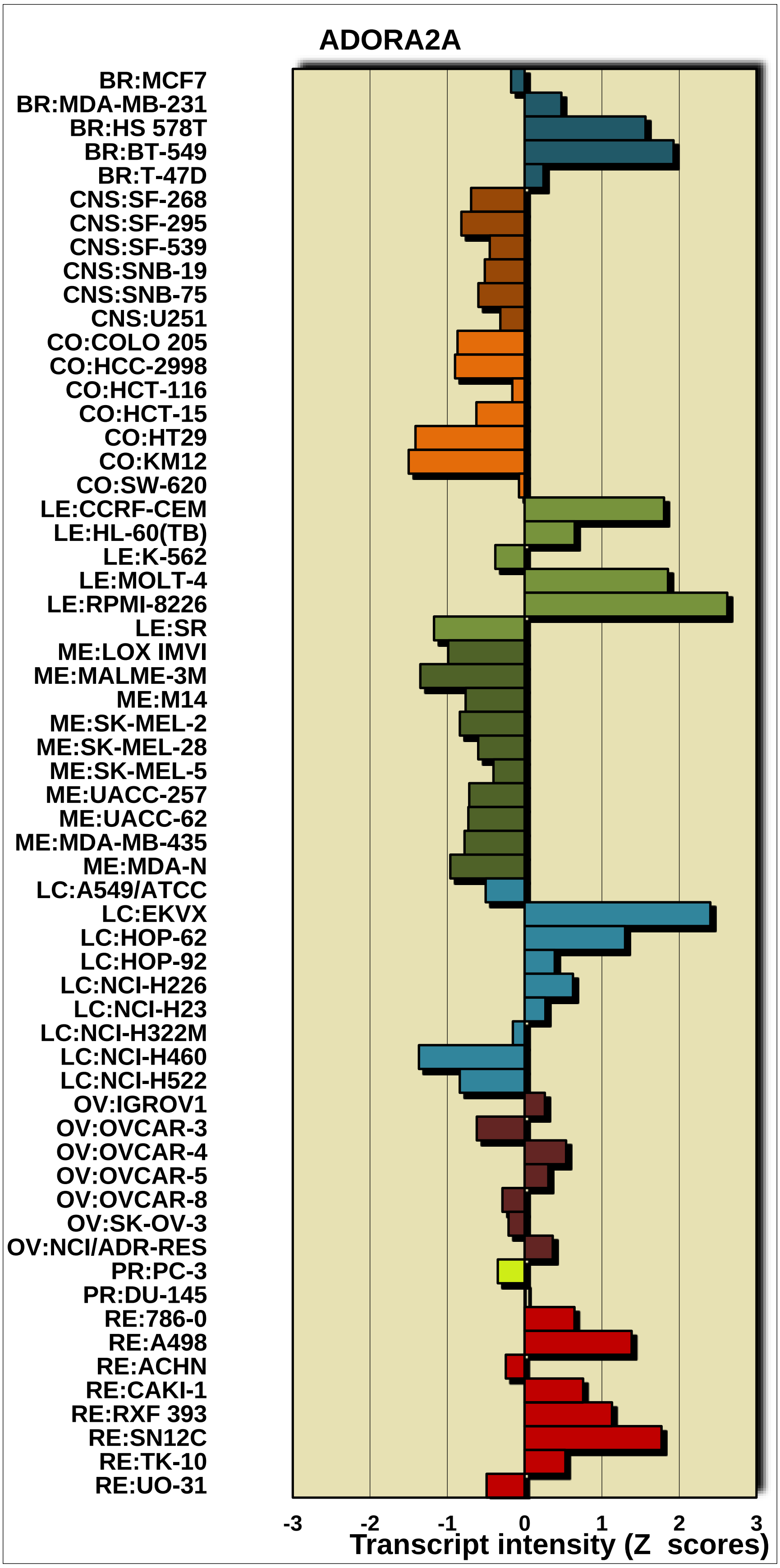
ADORA2A
BR:MCF7
BR:MDA-MB-231
BR:HS 578T
BR:BT-549
BR:T-47D
CNS:SF-268
CNS:SF-295
CNS:SF-539
CNS:SNB-19
CNS:SNB-75
CNS:U251
CO:COLO 205
CO:HCC-2998
CO:HCT-116
CO:HCT-15
CO:HT29
CO:KM12
CO:SW-620
LE:CCRF-CEM
LE:HL-60(TB)
LE:K-562
LE:MOLT-4
LE:RPMI-8226
LE:SR
ME:LOX IMVI
ME:MALME-3M
ME:M14
ME:SK-MEL-2
ME:SK-MEL-28
ME:SK-MEL-5
ME:UACC-257
ME:UACC-62
ME:MDA-MB-435
ME:MDA-N
LC:A549/ATCC
LC:EKVX
LC:HOP-62
LC:HOP-92
LC:NCI-H226
LC:NCI-H23
LC:NCI-H322M
LC:NCI-H460
LC:NCI-H522
OV:IGROV1
OV:OVCAR-3
OV:OVCAR-4
OV:OVCAR-5
OV:OVCAR-8
OV:SK-OV-3
OV:NCI/ADR-RES
PR:PC-3
PR:DU-145
RE:786-0
RE:A498
RE:ACHN
RE:CAKI-1
RE:RXF 393
RE:SN12C
RE:TK-10
RE:UO-31
-3
-2
-1
0
1
2
3
Transcript intensity (Z scores)

19.1 Table S17: Average transcript intensity z scores ($A_{2A}AR$) and GI_{50} for **1**

**Cell linesA_{2A}AR**	**Z scores**	Average GI_{50} (nM)
**BR:MCF7**	-0.175	46.35
**BR:MDA-MB-231**	0.476	141.00
**BR:HS 578T**	1.564	48.20
**BR:BT-549**	1.927	320.70
**BR:T-47D**	0.244	55.00
**CNS:SF-268**	-0.692	238.00
**CNS:SF-295**	-0.818	36.50
**CNS:SF-539**	-0.451	38.20
**CNS:SNB-19**	-0.515	70.10
**CNS:SNB-75**	-0.598	40.30
**CNS:U251**	-0.314	96.20
**CO:COLO 205**	-0.868	51.80
**CO:HCC-2998**	-0.9	213.00
**CO:HCT-116**	-0.159	66.80
**CO:HCT-15**	-0.624	70.50
**CO:HT29**	-1.412	49.00
**CO:KM12**	-1.5	67.30
**CO:SW-620**	-0.073	48.60
**LE:CCRF-CEM**	1.804	141.10
**LE:HL-60(TB)**	0.648	103.10
**LE:K-562**	-0.379	37.50
**LE:MOLT-4**	1.855	475.50
**LE:RPMI-8226**	2.622	255.50
**LE:SR**	-1.172	74.20
**ME:LOX IMVI**	-0.988	80.30
**ME:MALME-3M**	-1.349	36.30
**ME:M14**	-0.763	71.30
**ME:SK-MEL-2**	-0.838	47.70
**ME:SK-MEL-28**	-0.6	223.90
**ME:SK-MEL-5**	-0.403	237.40
**ME:UACC-257**	-0.716	0.00
**ME:UACC-62**	-0.728	50.30
**ME:MDA-MB-435**	-0.777	18.70
**ME:MDA-N**	-0.961	0.00
**LC:A549/ATCC**	-0.504	63.10
**LC:EKVX**	2.403	0.00

**LC:HOP-62**	1.3	110.40
**LC:HOP-92**	0.39	47.90
**LC:NCI-H226**	0.626	102.40
**LC:NCI-H23**	0.269	169.10
**LC:NCI-H322M**	-0.152	305.60
**LC:NCI-H460**	-1.367	40.80
**LC:NCI-H522**	-0.839	22.90
**OV:IGROV1**	0.262	196.30
**OV:OVCAR-3**	-0.619	48.90
**OV:OVCAR-4**	0.537	604.50
**OV:OVCAR-5**	0.307	307.50
**OV:OVCAR-8**	-0.287	279.00
**OV:SK-OV-3**	-0.208	60.60
**OV:NCI/ADR-RES**	0.364	46.30
**PR:PC-3**	-0.347	53.50
**PR:DU-145**	0.013	151.90
**RE:786-0**	0.645	103.30
**RE:A498**	1.385	23.20
**RE:ACHN**	-0.243	83.70
**RE:CAKI-1**	0.758	46.10
**RE:RXF 393**	1.13	41.80
**RE:SN12C**	1.771	142.00
**RE:TK-10**	0.527	0.00
**RE:UO-31**	-0.492	73.10

20 CXCR4

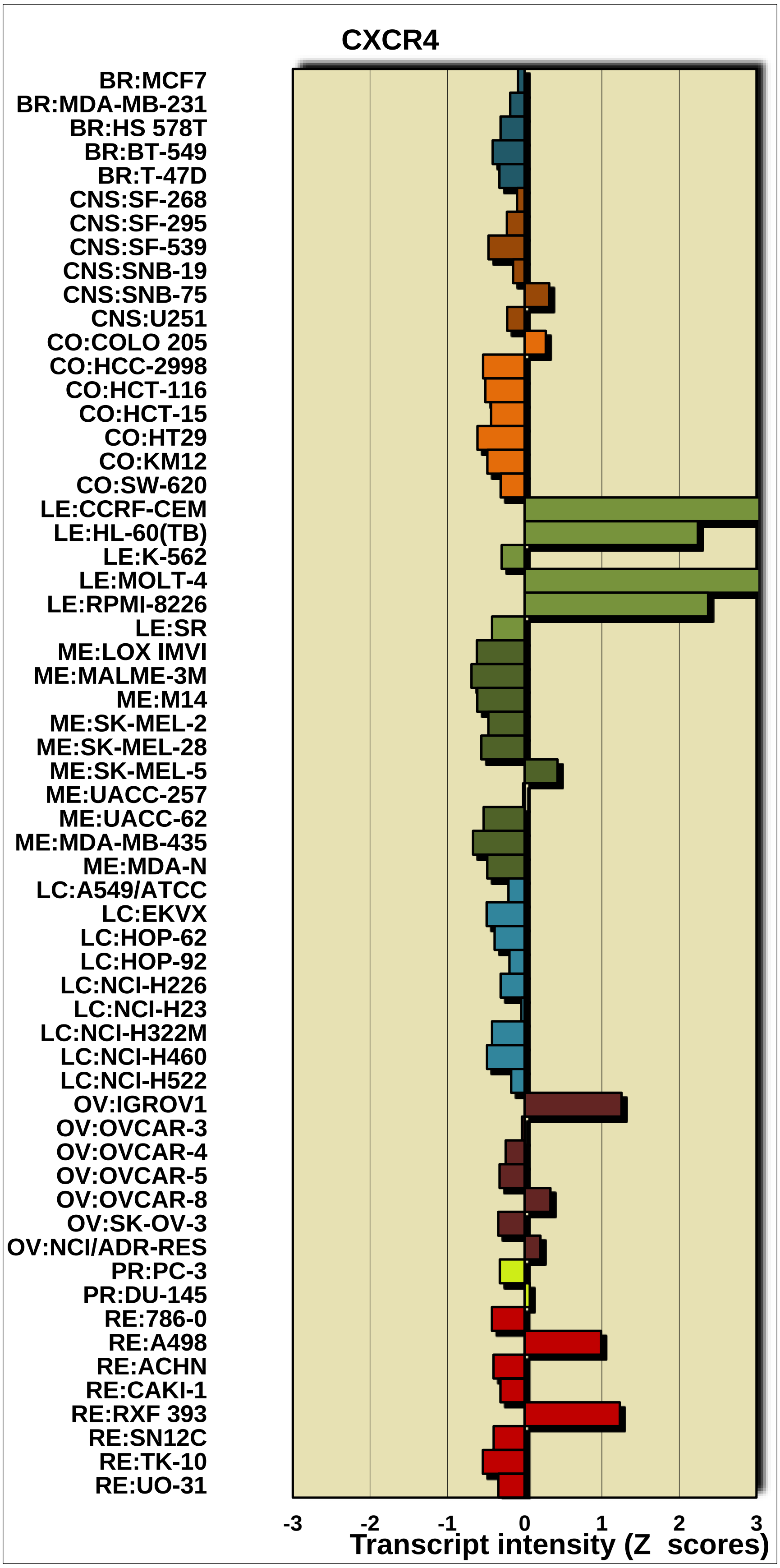
CXCR4
BR:MCF7
BR:MDA-MB-231
BR:HS 578T
BR:BT-549
BR:T-47D
CNS:SF-268
CNS:SF-295
CNS:SF-539
CNS:SNB-19
CNS:SNB-75
CNS:U251
CO:COLO 205
CO:HCC-2998
CO:HCT-116
CO:HCT-15
CO:HT29
CO:KM12
CO:SW-620
LE:CCRF-CEM
LE:HL-60(TB)
LE:K-562
LE:MOLT-4
LE:RPMI-8226
LE:SR
ME:LOX IMVI
ME:MALME-3M
ME:M14
ME:SK-MEL-2
ME:SK-MEL-28
ME:SK-MEL-5
ME:UACC-257
ME:UACC-62
ME:MDA-MB-435
ME:MDA-N
LC:A549/ATCC
LC:EKVX
LC:HOP-62
LC:HOP-92
LC:NCI-H226
LC:NCI-H23
LC:NCI-H322M
LC:NCI-H460
LC:NCI-H522
OV:IGROV1
OV:OVCAR-3
OV:OVCAR-4
OV:OVCAR-5
OV:OVCAR-8
OV:SK-OV-3
OV:NCI/ADR-RES
PR:PC-3
PR:DU-145
RE:786-0
RE:A498
RE:ACHN
RE:CAKI-1
RE:RXF 393
RE:SN12C
RE:TK-10
RE:UO-31
-3
-2
-1
0
1
2
3
Transcript intensity (Z scores)

20.1 Table S18: Average transcript intensity z scores (CXCR4) and GI_{50} for **1**

**Cell lines** ^{CXCR4}	**Z scores**	Average GI_{50} (nM)
**BR:MCF7**	-0.086	46.35
**BR:MDA-MB-231**	-0.186	141.00
**BR:HS 578T**	-0.311	48.20
**BR:BT-549**	-0.413	320.70
**BR:T-47D**	-0.326	55.00
**CNS:SF-268**	-0.098	238.00
**CNS:SF-295**	-0.229	36.50
**CNS:SF-539**	-0.467	38.20
**CNS:SNB-19**	-0.149	70.10
**CNS:SNB-75**	0.319	40.30
**CNS:U251**	-0.226	96.20
**CO:COLO 205**	0.274	51.80
**CO:HCC-2998**	-0.537	213.00
**CO:HCT-116**	-0.508	66.80
**CO:HCT-15**	-0.433	70.50
**CO:HT29**	-0.61	49.00
**CO:KM12**	-0.482	67.30
**CO:SW-620**	-0.31	48.60
**LE:CCRF-CEM**	3.474	141.10
**LE:HL-60(TB)**	2.241	103.10
**LE:K-562**	-0.297	37.50
**LE:MOLT-4**	3.615	475.50
**LE:RPMI-8226**	2.374	255.50
**LE:SR**	-0.421	74.20
**ME:LOX IMVI**	-0.618	80.30
**ME:MALME-3M**	-0.687	36.30
**ME:M14**	-0.612	71.30
**ME:SK-MEL-2**	-0.469	47.70
**ME:SK-MEL-28**	-0.56	223.90
**ME:SK-MEL-5**	0.426	237.40
**ME:UACC-257**	-0.018	0.00
**ME:UACC-62**	-0.53	50.30
**ME:MDA-MB-435**	-0.667	18.70
**ME:MDA-N**	-0.483	0.00
**LC:A549/ATCC**	-0.209	63.10
**LC:EKVX**	-0.491	0.00
**LC:HOP-62**	-0.388	110.40

**LC:HOP-92**	-0.196	47.90
**LC:NCI-H226**	-0.31	102.40
**LC:NCI-H23**	-0.044	169.10
**LC:NCI-H322M**	-0.421	305.60
**LC:NCI-H460**	-0.486	40.80
**LC:NCI-H522**	-0.174	22.90
**OV:IGROV1**	1.254	196.30
**OV:OVCAR-3**	-0.035	48.90
**OV:OVCAR-4**	-0.245	604.50
**OV:OVCAR-5**	-0.324	307.50
**OV:OVCAR-8**	0.334	279.00
**OV:SK-OV-3**	-0.341	60.60
**OV:NCI/ADR-RES**	0.205	46.30
**PR:PC-3**	-0.321	53.50
**PR:DU-145**	0.067	151.90
**RE:786-0**	-0.423	103.30
**RE:A498**	0.99	23.20
**RE:ACHN**	-0.403	83.70
**RE:CAKI-1**	-0.313	46.10
**RE:RXF 393**	1.231	41.80
**RE:SN12C**	-0.401	142.00
**RE:TK-10**	-0.54	0.00
**RE:UO-31**	-0.341	73.10

21 GPR35

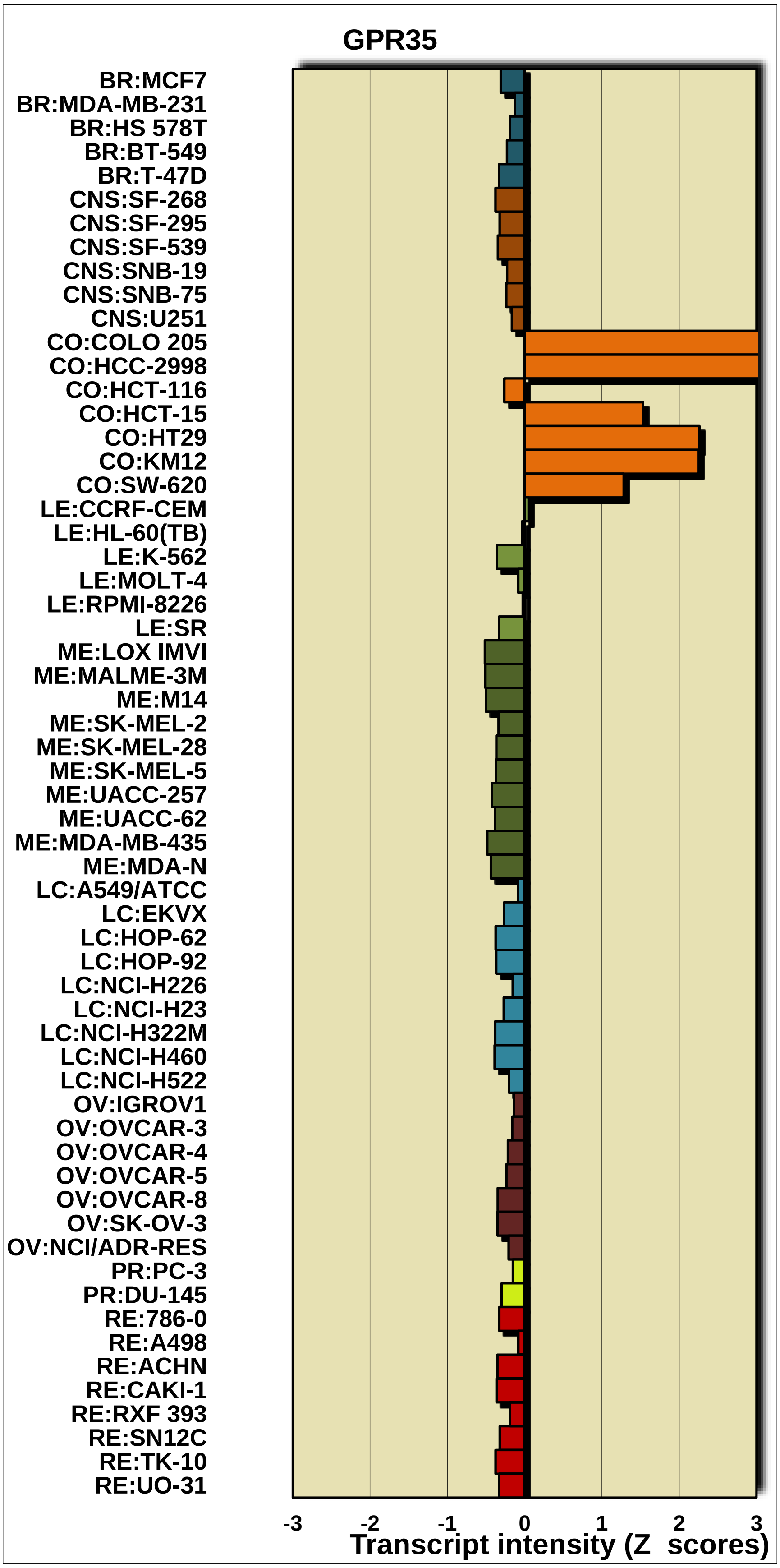
GPR35
BR:MCF7
BR:MDA-MB-231
BR:HS 578T
BR:BT-549
BR:T-47D
CNS:SF-268
CNS:SF-295
CNS:SF-539
CNS:SNB-19
CNS:SNB-75
CNS:U251
CO:COLO 205
CO:HCC-2998
CO:HCT-116
CO:HCT-15
CO:HT29
CO:KM12
CO:SW-620
LE:CCRF-CEM
LE:HL-60(TB)
LE:K-562
LE:MOLT-4
LE:RPMI-8226
LE:SR
ME:LOX IMVI
ME:MALME-3M
ME:M14
ME:SK-MEL-2
ME:SK-MEL-28
ME:SK-MEL-5
ME:UACC-257
ME:UACC-62
ME:MDA-MB-435
ME:MDA-N
LC:A549/ATCC
LC:EKVX
LC:HOP-62
LC:HOP-92
LC:NCI-H226
LC:NCI-H23
LC:NCI-H322M
LC:NCI-H460
LC:NCI-H522
OV:IGROV1
OV:OVCAR-3
OV:OVCAR-4
OV:OVCAR-5
OV:OVCAR-8
OV:SK-OV-3
OV:NCI/ADR-RES
PR:PC-3
PR:DU-145
RE:786-0
RE:A498
RE:ACHN
RE:CAKI-1
RE:RXF 393
RE:SN12C
RE:TK-10
RE:UO-31
-3
-2
-1
0
1
2
3
Transcript intensity (Z scores)

21.1 TableS19: Average transcript intensity z scores (GPR35) and GI_{50} for 1

**Cell lines** GPR35	**Z scores**	Average GI_{50} (nM)
**BR:MCF7**	-0.309	46.35
**BR:MDA-MB-231**	-0.127	141.00
**BR:HS 578T**	-0.19	48.20
**BR:BT-549**	-0.228	320.70
**BR:T-47D**	-0.329	55.00
**CNS:SF-268**	-0.377	238.00
**CNS:SF-295**	-0.323	36.50
**CNS:SF-539**	-0.346	38.20
**CNS:SNB-19**	-0.227	70.10
**CNS:SNB-75**	-0.237	40.30
**CNS:U251**	-0.165	96.20
**CO:COLO 205**	4.056	51.80
**CO:HCC-2998**	3.537	213.00
**CO:HCT-116**	-0.262	66.80
**CO:HCT-15**	1.532	70.50
**CO:HT29**	2.262	49.00
**CO:KM12**	2.253	67.30
**CO:SW-620**	1.282	48.60
**LE:CCRF-CEM**	0.052	141.10
**LE:HL-60(TB)**	-0.033	103.10
**LE:K-562**	-0.361	37.50
**LE:MOLT-4**	-0.082	475.50
**LE:RPMI-8226**	-0.024	255.50
**LE:SR**	-0.33	74.20
**ME:LOX IMVI**	-0.514	80.30
**ME:MALME-3M**	-0.507	36.30
**ME:M14**	-0.499	71.30
**ME:SK-MEL-2**	-0.337	47.70
**ME:SK-MEL-28**	-0.365	223.90
**ME:SK-MEL-5**	-0.372	237.40
**ME:UACC-257**	-0.424	0.00
**ME:UACC-62**	-0.383	50.30
**ME:MDA-MB-435**	-0.483	18.70
**ME:MDA-N**	-0.437	0.00
**LC:A549/ATCC**	-0.084	63.10
**LC:EKVX**	-0.264	0.00
**LC:HOP-62**	-0.375	110.40

**LC:HOP-92**	-0.367	47.90
**LC:NCI-H226**	-0.154	102.40
**LC:NCI-H23**	-0.27	169.10
**LC:NCI-H322M**	-0.38	305.60
**LC:NCI-H460**	-0.388	40.80
**LC:NCI-H522**	-0.202	22.90
**OV:IGROV1**	-0.138	196.30
**OV:OVCAR-3**	-0.16	48.90
**OV:OVCAR-4**	-0.216	604.50
**OV:OVCAR-5**	-0.235	307.50
**OV:OVCAR-8**	-0.348	279.00
**OV:SK-OV-3**	-0.35	60.60
**OV:NCI/ADR-RES**	-0.207	46.30
**PR:PC-3**	-0.152	53.50
**PR:DU-145**	-0.297	151.90
**RE:786-0**	-0.327	103.30
**RE:A498**	-0.081	23.20
**RE:ACHN**	-0.352	83.70
**RE:CAKI-1**	-0.362	46.10
**RE:RXF 393**	-0.19	41.80
**RE:SN12C**	-0.321	142.00
**RE:TK-10**	-0.376	0.00
**RE:UO-31**	-0.332	73.10

22 NPSRB1

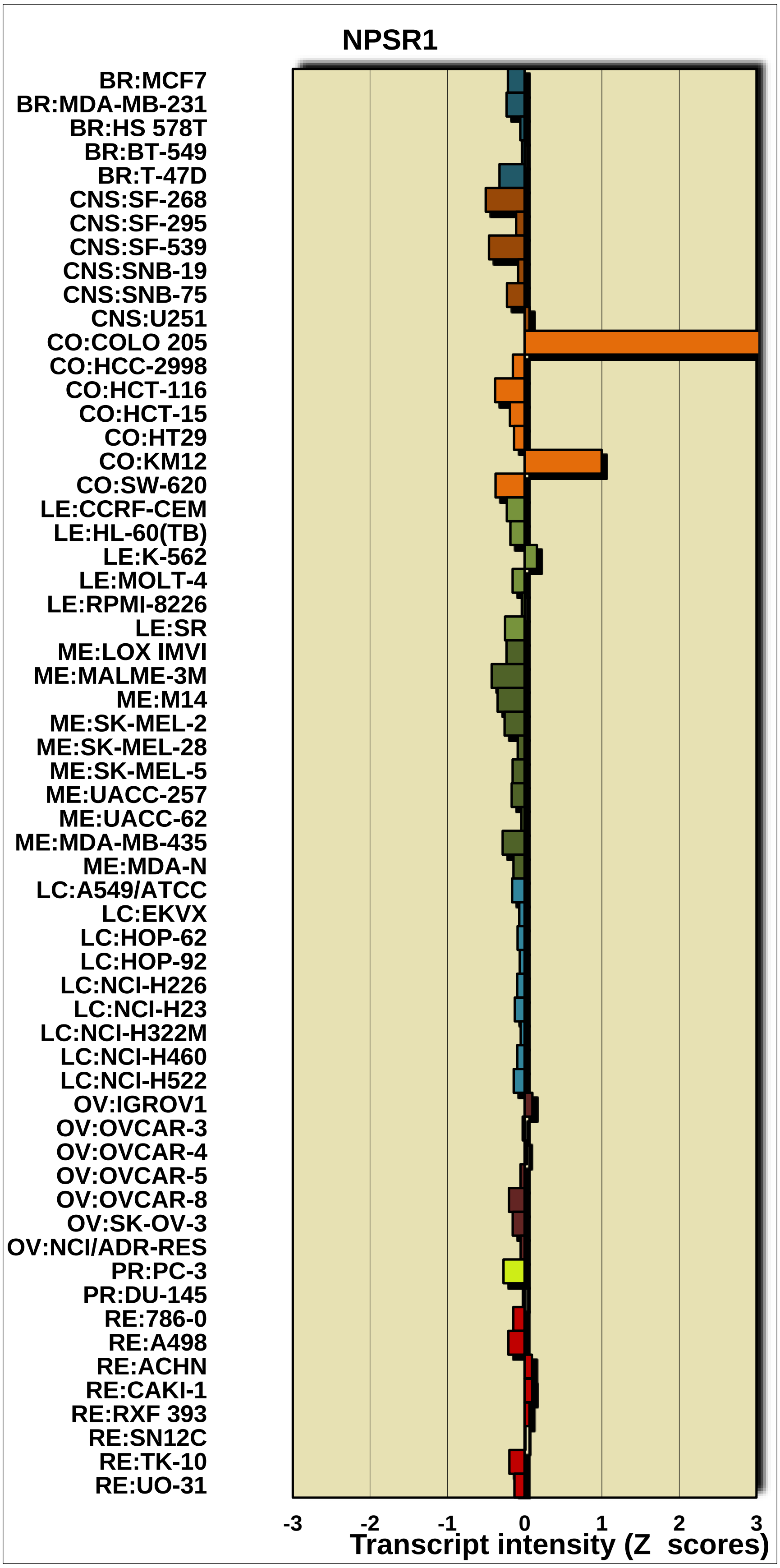
NPSR1
BR:MCF7
BR:MDA-MB-231
BR:HS 578T
BR:BT-549
BR:T-47D
CNS:SF-268
CNS:SF-295
CNS:SF-539
CNS:SNB-19
CNS:SNB-75
CNS:U251
CO:COLO 205
CO:HCC-2998
CO:HCT-116
CO:HCT-15
CO:HT29
CO:KM12
CO:SW-620
LE:CCRF-CEM
LE:HL-60(TB)
LE:K-562
LE:MOLT-4
LE:RPMI-8226
LE:SR
ME:LOX IMVI
ME:MALME-3M
ME:M14
ME:SK-MEL-2
ME:SK-MEL-28
ME:SK-MEL-5
ME:UACC-257
ME:UACC-62
ME:MDA-MB-435
ME:MDA-N
LC:A549/ATCC
LC:EKVX
LC:HOP-62
LC:HOP-92
LC:NCI-H226
LC:NCI-H23
LC:NCI-H322M
LC:NCI-H460
LC:NCI-H522
OV:IGROV1
OV:OVCAR-3
OV:OVCAR-4
OV:OVCAR-5
OV:OVCAR-8
OV:SK-OV-3
OV:NCI/ADR-RES
PR:PC-3
PR:DU-145
RE:786-0
RE:A498
RE:ACHN
RE:CAKI-1
RE:RXF 393
RE:SN12C
RE:TK-10
RE:UO-31
-3
-2
-1
0
1
2
3
Transcript intensity (Z scores)

22.1 **Table S20:** Average transcript intensity z scores (NPSRB1) and GI_{50} for **1**

**Cell lines ^{NPSR1B}**	**Z scores**	Average GI_{50} (nM)
**BR:MCF7**	-0.216	46.35
**BR:MDA-MB-231**	-0.233	141.00
**BR:HS 578T**	-0.056	48.20
**BR:BT-549**	-0.035	320.70
**BR:T-47D**	-0.325	55.00
**CNS:SF-268**	-0.503	238.00
**CNS:SF-295**	-0.11	36.50
**CNS:SF-539**	-0.461	38.20
**CNS:SNB-19**	-0.083	70.10
**CNS:SNB-75**	-0.228	40.30
**CNS:U251**	0.063	96.20
**CO:COLO 205**	6.804	51.80
**CO:HCC-2998**	-0.152	213.00
**CO:HCT-116**	-0.381	66.80
**CO:HCT-15**	-0.19	70.50
**CO:HT29**	-0.136	49.00
**CO:KM12**	0.997	67.30
**CO:SW-620**	-0.376	48.60
**LE:CCRF-CEM**	-0.23	141.10
**LE:HL-60(TB)**	-0.184	103.10
**LE:K-562**	0.159	37.50
**LE:MOLT-4**	-0.155	475.50
**LE:RPMI-8226**	-0.036	255.50
**LE:SR**	-0.253	74.20
**ME:LOX IMVI**	-0.234	80.30
**ME:MALME-3M**	-0.426	36.30
**ME:M14**	-0.349	71.30
**ME:SK-MEL-2**	-0.259	47.70
**ME:SK-MEL-28**	-0.089	223.90
**ME:SK-MEL-5**	-0.155	237.40
**ME:UACC-257**	-0.167	0.00
**ME:UACC-62**	-0.043	50.30
**ME:MDA-MB-435**	-0.284	18.70
**ME:MDA-N**	-0.144	0.00
**LC:A549/ATCC**	-0.162	63.10
**LC:EKVX**	-0.069	0.00
**LC:HOP-62**	-0.091	110.40

**LC:HOP-92**	-0.063	47.90
**LC:NCI-H226**	-0.096	102.40
**LC:NCI-H23**	-0.127	169.10
**LC:NCI-H322M**	-0.05	305.60
**LC:NCI-H460**	-0.095	40.80
**LC:NCI-H522**	-0.141	22.90
**OV:IGROV1**	0.101	196.30
**OV:OVCAR-3**	-0.025	48.90
**OV:OVCAR-4**	0.033	604.50
**OV:OVCAR-5**	-0.053	307.50
**OV:OVCAR-8**	-0.202	279.00
**OV:SK-OV-3**	-0.154	60.60
**OV:NCI/ADR-RES**	-0.051	46.30
**PR:PC-3**	-0.273	53.50
**PR:DU-145**	-0.023	151.90
**RE:786-0**	-0.147	103.30
**RE:A498**	-0.209	23.20
**RE:ACHN**	0.095	83.70
**RE:CAKI-1**	0.1	46.10
**RE:RXF 393**	0.062	41.80
**RE:SN12C**	0.01	142.00
**RE:TK-10**	-0.197	0.00
**RE:UO-31**	-0.132	73.10
